# Genome-wide screen reveals glycerol-induced aminoglycoside potentiation against *Staphylococcus aureus* via boosting GlpK-initiated energy metabolism

**DOI:** 10.1128/aac.00938-25

**Published:** 2025-10-23

**Authors:** Yaqin Xing, Mengmeng Bian, Xuebing Huang, Boyan Lv, Zhijie Huang, Xianzhang Jiang, Weiya Huang, Huping Xue, Hangyu Zhao, Jianfeng Huang, Xinmiao Fu

**Affiliations:** 1 Provincial University Key Laboratory of Cellular Stress Response and Metabolic Regulation, College of Life Sciences, Fujian Normal University 12425, Fuzhou, Fujian, China; 2 Institute of Precision Medicine, Fujian Provincial Hospital, Fuzhou University Affiliated Provincial Hospital 117861, Fuzhou, Fujian, China; 3 Department of Animal Science and Technology, University of Northwest A&F 12469, Yangling, Shaanxi, China; The Peter Doherty Institute for Infection and Immunity, Melbourne, Victoria, Australia

**Keywords:** antibiotic resistance, bacterial persister, MRSA, aminoglycoside, glycerol, energy metabolism, PMF

## Abstract

Potentiation of existing antibiotics represents a promising strategy for combating the global health crisis of antibiotic resistance. Here we report that 1 min co-treatment with glycerol markedly enhances aminoglycoside efficacy against stationary-phase *Staphylococcus aureus* cells. This rapid combined treatment also effectively eliminates *S. aureus* biofilms and persister cells, methicillin-resistant *S. aureus* clinical isolates *in vitro*, and *S. aureus* in a mouse skin infection model. The potentiation is achieved through rapid enhancement of proton motive force-dependent aminoglycoside uptake. Atmospheric and room temperature plasma (ARTP)-based genome-wide mutagenesis screens further reveal the genetic basis of this potentiation, highlighting a central role for glycerol kinase (GlpK). Mechanistically, GlpK initiates glycerol catabolism and boosts cellular energy metabolism, thereby increasing proton motive force-driven aminoglycoside uptake. Unlike widely reported long-term adjuvant treatments, our approach offers an immediate potentiation strategy and may open avenues to develop glycerol as an aminoglycoside adjuvant, given its “generally recognized as safe” characteristic. Our study also illustrates the potential of ARTP-based genome-wide mutagenesis in antibiotic resistance and stress biology research.

## INTRODUCTION

The discovery and application of antibiotics are among the most transformative achievements in modern medicine, extending the human lifespan by over 20 years (1). In 2019 alone, however, bacterial infections caused by 33 major pathogens accounted for 7.7 million deaths worldwide, representing 13.6% of global mortality and establishing bacterial infections as the second leading cause of death globally that year (2). Compounding this crisis, the spread of antibiotic resistance, first documented by Alexander Fleming in the 1930s (3), has escalated into a critical public health crisis, resulting in 4.95 and 1.27 million deaths in 2019 that are, respectively, associated with and attributable to the resistance (4). Hence, this alarming trajectory demands urgent, coordinated global action through multifaceted strategies including (i) accelerated development of novel antibiotics (5, 6); (ii) optimized stewardship of existing antibiotics (7, 8); and (iii) advancement of alternative therapeutic approaches such as phage therapy and immunomodulation (9, 10). Furthermore, the clinical challenge extends beyond resistance mechanisms to include bacterial persistence—a phenotypic tolerance state enabling bacterial survival during antibiotic exposure (11–13). This adaptive strategy not only leads to antibiotic tolerance but may also facilitate the evolution of antibiotic resistance (14), emphasizing the need for comprehensive approaches targeting both resistant and persistent bacterial populations.

While the development of novel antibiotics with distinct mechanisms of action remains the preferred strategy for addressing antimicrobial resistance, current preclinical and clinical pipelines demonstrate insufficient capacity to sustainably counter this evolving threat (10). Furthermore, only a few novel antibiotic classes have reached clinical application since the 1990s (6), reflecting both scientific challenges in target discovery and economic disincentives in pharmaceutical development. This paradigm underscores the critical importance of optimizing existing antimicrobial agents through pharmacological enhancement—an approach leveraging established safety profiles, pharmacokinetic understanding, and pharmacodynamic properties to reduce developmental costs (15). Given the very complicated interaction between bacterial metabolism and antibiotic efficacy (11), multiple metabolites, including glucose, mannitol, alanine, pyruvate, glycerol, formate, fructose, cis-2-decenoic acid, N-acetyl-D-glucosamine, and metformin, have been reported to be capable of potentiating major antibiotic classes such as aminoglycosides, fluoroquinolones, β-lactams, and tetracyclines (8, 16–26), which are summarized as a metabolic state-driven, nutrient-based approach (20).

Among bactericidal antibiotics, aminoglycosides emerge as particularly amenable to potentiation strategies because both their bacterial uptake and downstream lethal actions are linked to cellular respiration, which can be regulated accordingly (27). However, conventional aminoglycoside potentiation approaches typically require multi-hour treatment durations (17, 18, 24). Our group recently demonstrated that 1 min physical interventions, including hypoionic shock (e.g., ion-free treatment via pure water) (28), heat shock (29), or freezing (30), can markedly enhance aminoglycoside efficacy against stationary-phase *Escherichia coli* cells. Notably, 1 min co-treatment with *n*-butanol or 5-methyl-indole enables aminoglycosides to achieve unprecedented four-log reductions against stationary-phase *Staphylococcus aureus* cells (31, 32) that are extremely tolerant to bactericidal antibiotics (33).

Building upon these findings, we aimed to develop a clinically translatable antibiotic potentiation strategy balancing biological safety with practical implementation. Our current work reveals that glycerol, a compound ubiquitously employed in food and pharmaceutical formulations, synergistically enhances aminoglycoside bactericidal activity against *S. aureus* through mere 1 min co-exposure. Utilizing a novel genome-wide mutagenesis screen tool, we systematically mapped the molecular pathways governing this glycerol-mediated potentiation. This proof-of-concept study not only demonstrates the potential of glycerol as an excellent aminoglycoside adjuvant but also illustrates atmospheric and room temperature plasma (ARTP) as a generally useful tool for antibiotic resistance research.

## RESULTS

### Characterization of glycerol-mediated aminoglycoside potentiation against stationary-phase *S. aureus*

**Fig 1:**
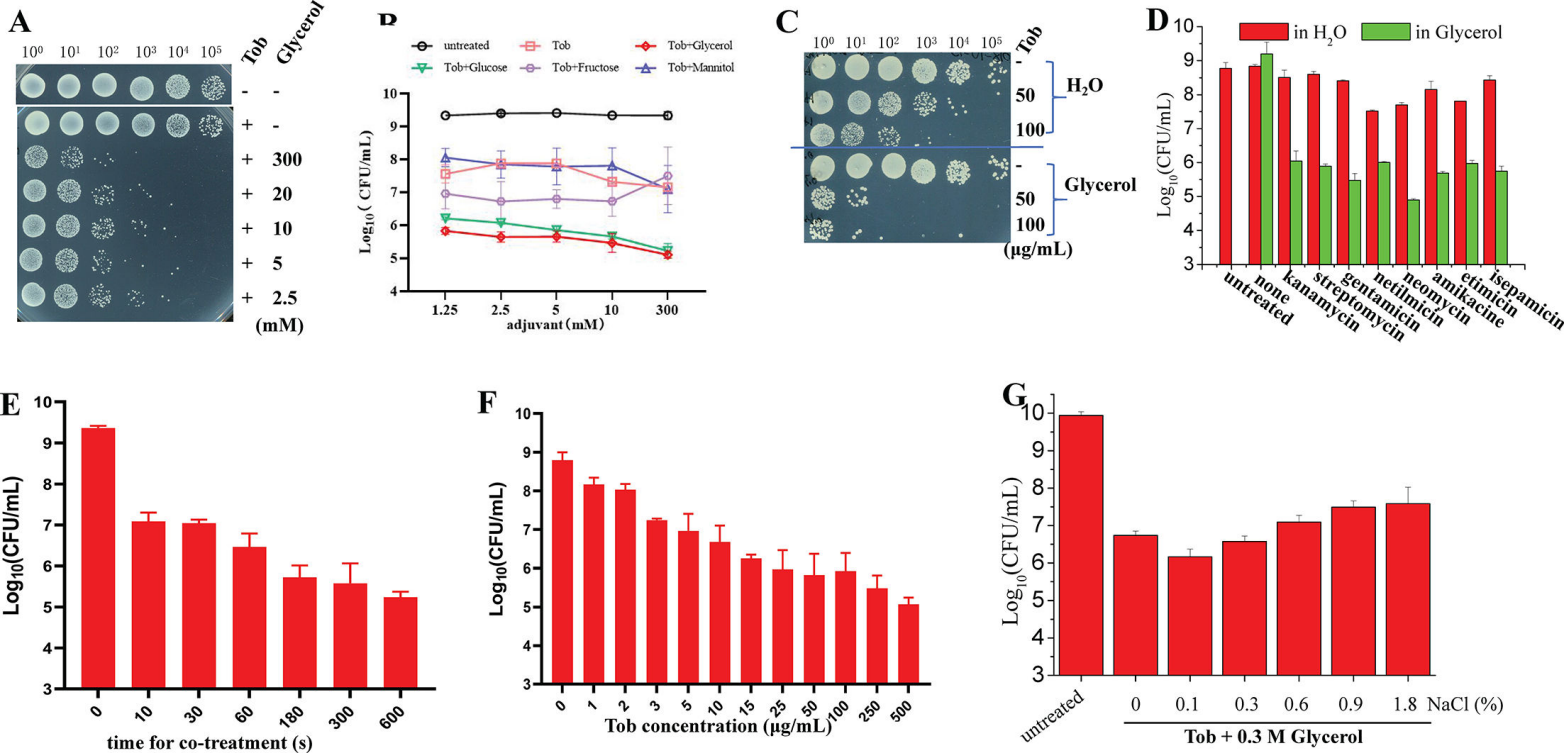
Rapid glycerol co-treatment enables aminoglycoside to eradicate stationary-phase *S. aureus* cells. (**A**) Survival of stationary-phase *S. aureus* cells following a 3 min treatment with 50 µg/mL tobramycin in the presence of increasing concentrations of glycerol. (**B**) Quantified survival of stationary-phase *S. aureus* cells following a 3 min treatment with 50 µg/mL tobramycin in the presence of various types of carbon sources. (**C**) Survival of exponential-phase *S. aureus* cells following a 3 min treatment with 50 or 100 µg/mL tobramycin as dissolved in pure water or 0.3 M glycerol solution. (**D**) Survival of stationary-phase *S. aureus* cells following a 3 min treatment with various types of aminoglycoside antibiotics as dissolved in pure water or 0.3 M glycerol solution. (**E, F, G**) Survival of stationary-phase *S. aureus* cells following a combined treatment with 50 µg/mL tobramycin plus 0.3 M glycerol for varying length of time (panel **E**), with increasing concentrations of tobramycin plus 0.3 M glycerol for 3 min (panel **F**), or with 50 µg/mL tobramycin plus 0.3 M glycerol for 3 min in the presence of indicated concentrations of NaCl (panel **G**). Data in panels **B**, **D**, **E**, **F**, and **G** represent the means ± SD of three replicates in one independent experiment.

While previous studies established that prolonged co-treatment (hours) with metabolic substrates like glucose, fructose, mannitol, and glycerol enhances aminoglycoside efficacy (17, 18, 23, 24, 27, 34), our prior work demonstrated that *n*-butanol achieves rapid aminoglycoside potentiation against *S. aureus* within minutes under hypoionic conditions (31). Building on this paradigm, we identified that 3 min hypoionic co-treatment (in pure water) with various carbon sources—excluding pyruvate—significantly enhanced tobramycin activity against stationary-phase *S. aureus* (Fig. S1A). Dose-response analysis revealed glycerol and glucose as superior potentiators compared to fructose and mannitol (Fig. 1A and B).

Given glucose’s potential limitations in clinical settings due to its association with diabetic complications and glycerol’s GRAS (generally recognized as safe) feature, we prioritized further characterization of glycerol. First, glycerol at low concentrations (from 0.05 mM to 1 mM) exhibited potentiation effects in a concentration-dependent manner (Fig. S1B), although its potentiation effect at >1 mM appeared constant (Fig. 1B). Second, such glycerol-induced tobramycin potentiation was also observed against exponential-phase *S. aureus* cells under hypoionic conditions (Fig. 1C). Third, glycerol was able to facilitate various types of aminoglycoside antibiotics to kill stationary-phase *S. aureus* cells (Fig. 1D), but had little potentiation effect toward β-lactams (represented by ampicillin and meropenem) and fluoroquinolones (represented by ofloxacin and ciprofloxacin) (Fig. S1C), indicating glycerol-induced potentiation is aminoglycoside-specific. In addition, the potentiation effect could be detected when the co-treatment duration is as short as 10 seconds (Fig. 1E) or when the concentration of tobramycin is as low as 1 µg/mL (Fig. 1F; note: minimal inhibition concentration [MIC], is 2 µg/mL). Notably, such glycerol-induced tobramycin potentiation was largely retained in the presence of excessive NaCl (Fig. 1G), in contrast to 5-methyl-indole- or *n*-butanol-induced aminoglycoside potentiation against *S. aureus* cells only occurring under hypoionic conditions as we reported earlier (31, 32). Nevertheless, the presence of excessive CaCl_2_ or MgCl_2_ could nearly abolish the potentiation effect of glycerol (Fig. S1D).

### Glycerol potentiates aminoglycosides killing of *S. aureus* biofilms and persisters, clinical isolates of methicillin*-*resistant *S. aureus* (MRSA), and *Staphylococcus epidermidis* cells

**Fig 2:**
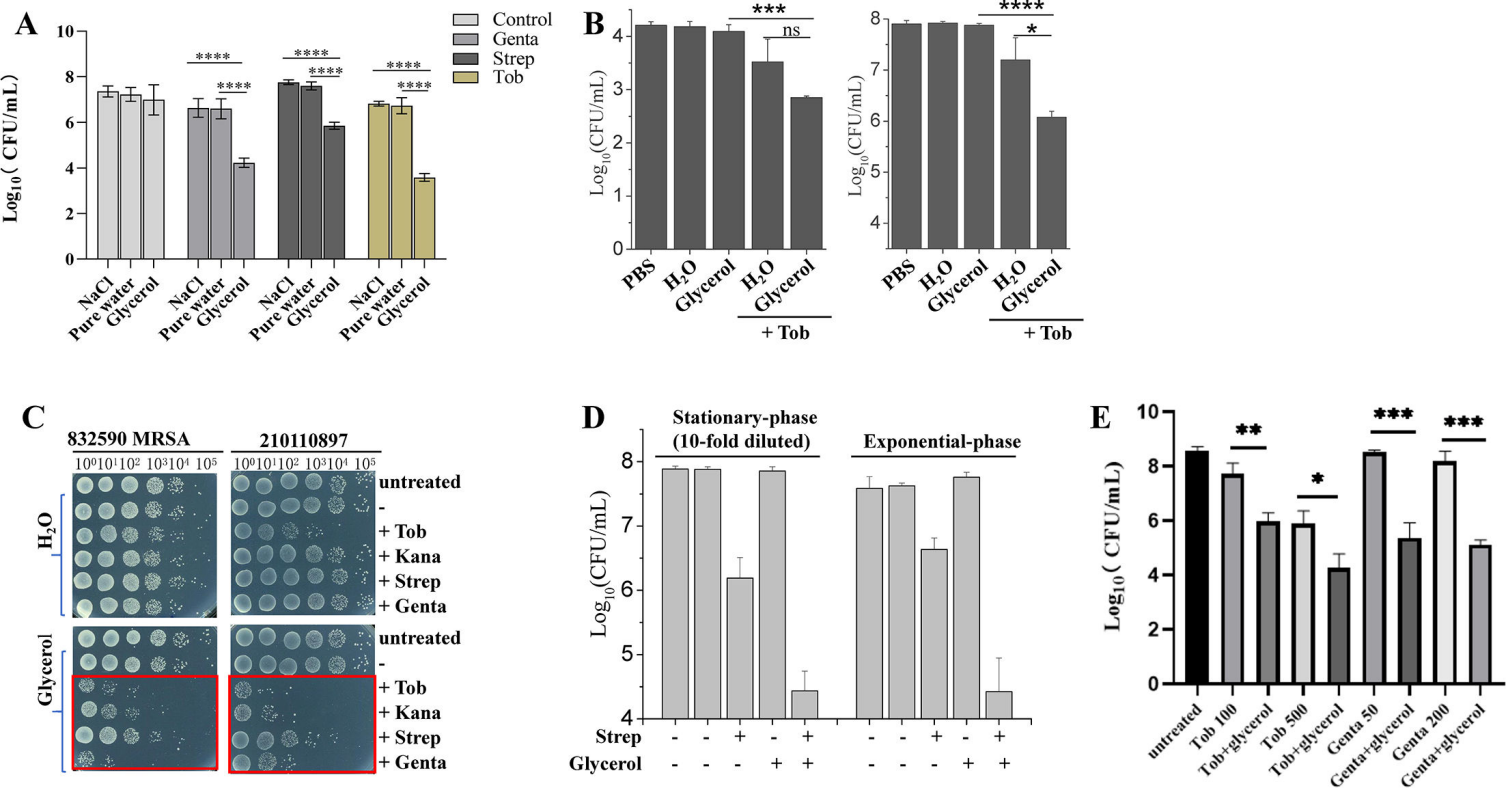
Glycerol enables aminoglycoside to rapidly kill *S. aureus* biofilms, persisters, and clinical isolates and MRSA cells. (**A**) Survival of *S. aureus* biofilms following a 3 min treatment with 50 µg/mL gentamicin, tobramycin, or streptomycin as dissolved in 0.9% NaCl solution, pure water, or 0.3 M glycerol solution. (**B**) Survival of *S. aureus* persisters following a 3 min treatment with 25 µg/mL tobramycin as dissolved in pure water or 0.3 M glycerol solution. Left part: exponential-phase *S. aureus* cells were pretreated with 25 µg/mL gentamicin for 3 h. Right part: stationary-phase *S. aureus* cells as cultured in Mueller Hinton Broth (MHB) medium were transferred into Yeast Nitrogen Base (YNB) medium for 5 h starvation. (**C**) Survival of typical clinical isolates of methicillin-resistant (left part) and methicillin-sensitive (right part) *S. aureus* cells in stationary phase following a 3 min treatment with tobramycin, kanamycin, streptomycin, or gentamicin as dissolved in pure water or 0.3 M glycerol solution. Results of other isolates are presented in Fig. S3 and S4A. (**D**) Survival of MRSA ATCC43300 strain (MIC is 50 µg/mL streptomycin, Fig. S4B) following a 3 min treatment with 20 µg/mL streptomycin as dissolved in pure water or 0.3 M glycerol solution. (**E**) Survival of stationary-phase *S. epidermidis cells* following a 3 min treatment with tobramycin or gentamicin as dissolved in pure water or 0.3 M glycerol solution. Data in panels **A**, **B**, **D**, and **E** represent the means ± SD of three replicates in one independent experiment. “*”, “**”, “***” and “****” indicate significance levels with *P* value less than 0.05, 0.01, 0.001 and 0.001, respectively, and “ns” indicates *P* > 0.05 (i.e., not significant).

Next, we examined whether glycerol could facilitate aminoglycosides to eradicate *S. aureus* biofilms, persisters, and clinical isolates that are potentially persistent and resistant to antibiotic therapy (35, 36). First, mature *S. aureus* biofilms were established through 24 h static culture in 24-well polystyrene plates, and 3 min treatment with tobramycin, gentamicin, or streptomycin revealed striking phase-dependent efficacy: glycerol-formulated aminoglycosides achieved around two-log biofilm reduction, while identical concentrations in NaCl or pure water showed negligible bactericidal activity (Fig. 2A). Second, spontaneous persisters were prepared by pretreating *S. aureus* exponential-phase cells with tobramycin for 3 h by referring to the growth curve (Fig. S2A) and time-killing assay (Fig. S2B), and such surviving persister cells (up to 0.01% of the population) were killed by 95% with tobramycin plus glycerol (Fig. 2B, left). We also generated starvation-induced *S. aureus* persisters (37, 38), which were effectively killed by 99% with the combined treatment (Fig. 2B, right).

We further examined MRSA, which has been placed in the WHO priority list of most dangerous bacterial pathogens (39). Of 18 multidrug-resistant MRSA strains (Table S2), 16 could be effectively killed by the combined treatment with at least one of the four aminoglycosides (i.e., tobramycin, kanamycin, streptomycin, or gentamicin) plus glycerol (left part of Fig. 2C and Fig. S3). Of five methicillin-sensitive but multidrug-resistant clinical strains of *S. aureus* (Table S3), four strains could be effectively killed by at least one of the four aminoglycosides plus glycerol (right part of Fig. 2C, and Fig. S4A). In addition, the MRSA ATCC43300 strain, which seemed to be resistant to streptomycin (Fig. S4B), was killed by sub-inhibitory concentration of streptomycin plus glycerol (Fig. 2D). Notably, glycerol could facilitate tobramycin and gentamicin to kill stationary-phase *S. epidermidis* cells as well (Fig. 2E), but it had little potentiation effect against other gram-positive bacteria, including *Streptococcus pyogenes*, *Lactococcus lactis, Enterococcus faecalis,* and *Micrococcus luteus* (Fig. S5).

### Glycerol potentiates tobramycin killing of *S. aureus* in a mouse skin infection model

**Fig 3:**
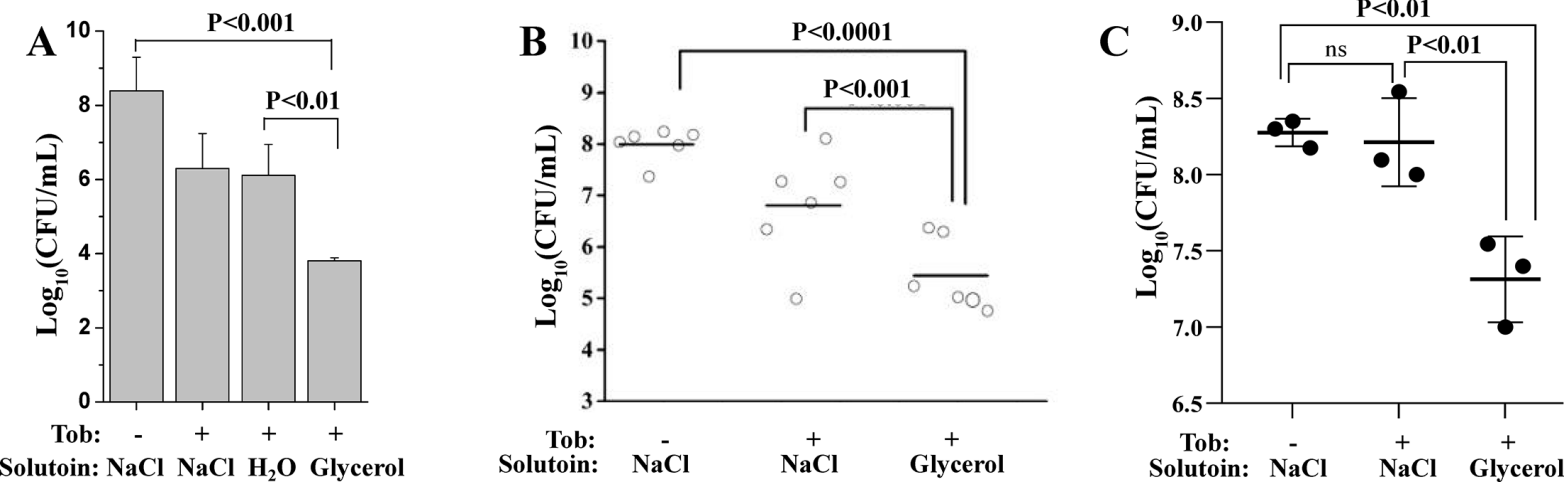
Glycerol potentiates tobramycin against *S. aureus* skin infection in mice. (**A**) Survival of 5 × 10^7^ *S. aureus* cells in stationary phase after the cells were plated on a piece of isolated mouse skin and subjected to the combined treatment with 100 µg/mL tobramycin or streptomycin as dissolved in 0.9% NaCl solution, pure water, or 0.3 M glycerol. Data represent the means ± SD of three replicates. (**B**) Survival of 5 × 10^7^ *S. aureus* cells from the wound of mice (*n* = 6) following a 3 min treatment with 50 µg/mL tobramycin as dissolved in NaCl solution or 0.3 M glycerol solution (refer to Fig. S6A). (**C**) Survival of *S. aureus* biofilms from the wound of mice following a 3 min treatment with 200 µg/mL tobramycin as dissolved in NaCl solution or 0.3 M glycerol solution (refer to Fig. S6B). Biofilms were generated by pre-seeding around 1 × 10^7^ cells in the wound of mice (*n* = 3) followed by wrapping and incubating for 48 h. Each circle in panels **B** and **C** represents colony-forming units (CFU) value from one mouse; averaged CFU of each group is shown as a short line. Significance levels are calculated by analysis of variance. ns, significance level with *P* value >0.05 (i.e., not significant).

As a proof of concept, we further demonstrated that glycerol enhanced the efficacy of aminoglycosides in eradicating *S. aureus* in a mouse model. Specifically, skin specimens were excised from euthanized mice and inoculated with stationary-phase *S. aureus* cells, followed by combined treatment with the antibiotic and glycerol. Cell survival assays revealed that tobramycin dissolved in glycerol solution reduced the viability of skin-adherent *S. aureus* by more than four orders of magnitude. In contrast, when the antibiotic was delivered in NaCl solution or pure water, the reduction was only approximately two orders of magnitude (Fig. 3A).

Second, full-thickness 1 cm² dorsal excisions were created in anesthetized mice and inoculated with *S. aureus* prior to therapeutic intervention (Fig. S6A, left). The tobramycin-glycerol combination achieved significant pathogen reduction (*P* < 0.001) compared to monotherapy controls (Fig. 3B, right of Fig. S6A). Building on *in vitro* biofilm eradication data (Fig. 2A) and dosage assay results (Fig. 1F), we established a mouse wound biofilm infection model (Fig. S6B) and showed that such *S. aureus* biofilms could be effectively eradicated by tobramycin plus glycerol but not by tobramycin in NaCl solution even at a concentration of 100× MIC (i.e., 200 µg/mL) (Fig. 3C), indicating good efficacy of the combined treatment against *S. aureus* biofilms with high antibiotic tolerance. Together, these observations demonstrate that glycerol facilitates tobramycin killing of *S. aureus* cells in the mouse skin infection model.

### Glycerol-induced tobramycin potentiation is achieved by enhancing proton motive force (PMF)-dependent antibiotic uptake

**Fig 4:**
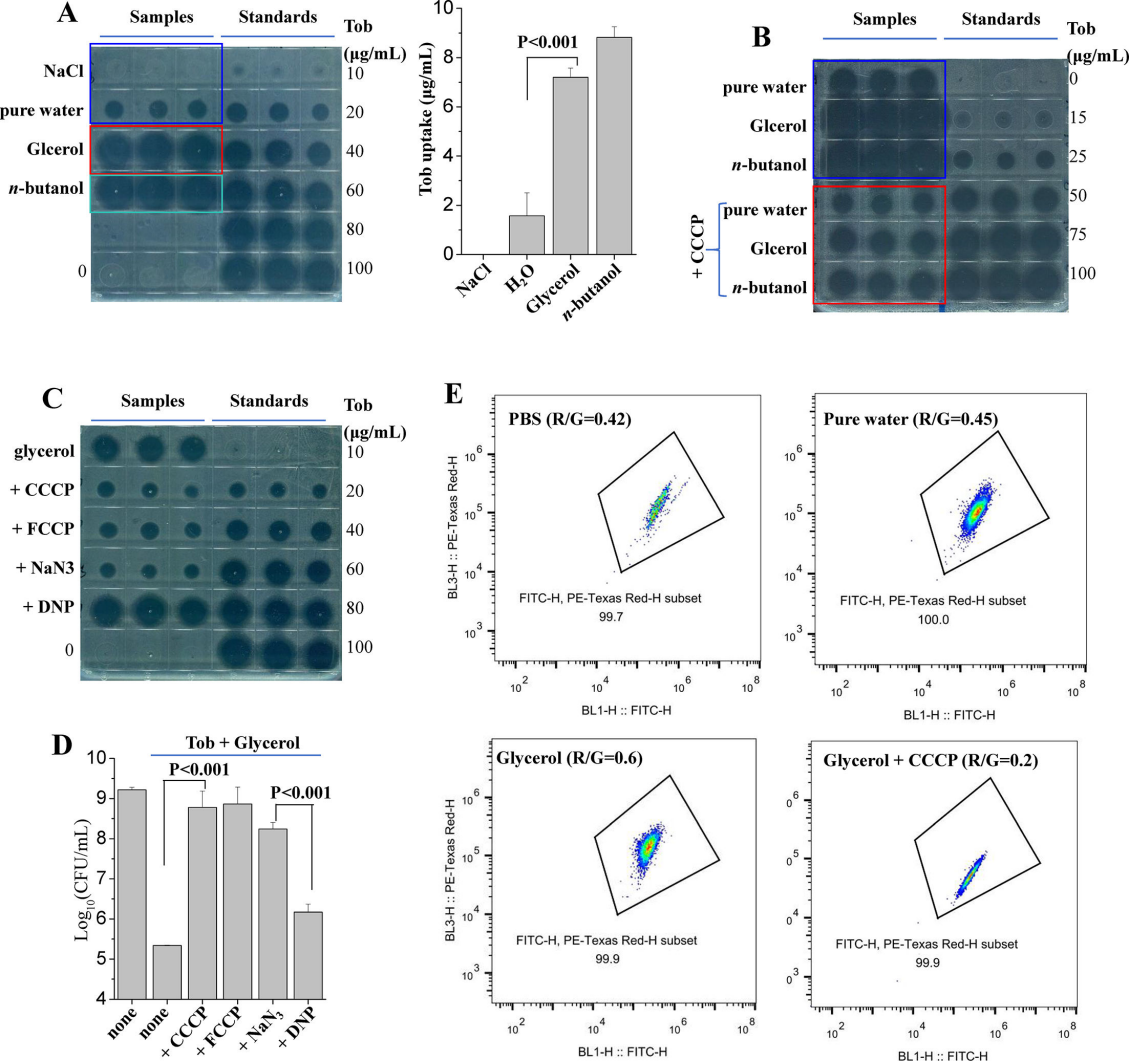
Rapid glycerol co-treatment enhances the bacterial uptake of tobramycin in a PMF-dependent manner. (**A, B, C**) Inhibition of *E. coli* cell growth on LB agar dishes by tobramycin extracted from stationary-phase *S. aureus* cells. Panel A: *S. aureus* cells were treated with 100 µg/mL tobramycin dissolved in 0.9% NaCl, pure water, 0.3 M glycerol, or 0.3 M *n-*butanol solution (right part: quantified uptake of tobramycin); panels B and C: *S. aureus* cells were pretreated with CCCP, FCCP, NaN_3_, or DNP for 1 h prior to a 3 min treatment with 100 µg/mL tobramycin plus 0.3 M glycerol. Tobramycin was extracted and then subjected to cell growth inhibition assay as described in the Materials and Methods section. (**D**) Survival of stationary-phase *S. aureus* cells after the treatment as described in panel C. (**E**) Results of a flow cytometric analysis of stationary-phase *S. aureus* cells after treatment with indicated buffers (phosphate-buffered saline, pure water, glycerol or glycerol plus CCCP). Relative PMF level is indicated by the ratio of red fluorescence to green fluorescence (R/G ratio).

We sought to uncover the molecular mechanism underlying rapid glycerol treatment-induced aminoglycoside potentiation. We previously reported that *n*-butanol- and hypoionic shock-aminoglycoside potentiation against *S. aureus* cells was achieved via enhancing the bacterial uptake of aminoglycoside (31, 37). Therefore, we measured tobramycin uptake of *S. aureus* cells by using tobramycin extraction coupled with a cell growth inhibition assay, a method we explored (30, 37). Our results indicate that tobramycin, as extracted from *S. aureus* cells that were pretreated with tobramycin plus glycerol, strongly suppressed the growth of pre-seeded *E. coli* cells on luria broth (LB) agar dishes (red frame in Fig. 4A), indicating a significant uptake of tobramycin. Consistently, the presence of *n*-butanol during pretreatment also led to strong cell growth inhibition (green frame), as we previously reported (31). By contrast, little and slight cell growth inhibition effects were observed if *S. aureus* cells were pretreated with tobramycin dissolved in NaCl solution and in pure water, respectively (blue frame). In addition, we also observed glycerol-enhanced tobramycin uptake by *S. aureus* biofilms (Fig. S7A). These observations thus demonstrate that rapid glycerol treatment is able to greatly enhance the bacterial uptake of tobramycin by *S. aureus* cells, which largely accounts for glycerol-enhanced tobramycin lethality.

It is well-known that the bacterial uptake of aminoglycosides under conventional treatment conditions (40) or upon metabolite stimulation (17, 18) depends on the PMF. We thus treated cells with the protonophore CCCP to dissipate PMF. Data showed that co-treatment with CCCP largely abolished such glycerol-enhanced tobramycin uptake by *S. aureus* cells (Fig. 4B) and also protected the cells from being killed by the combined treatment in a concentration-dependent manner (Fig. S7B). Consistently, FCCP, a functional analog of CCCP, was able to effectively suppress both glycerol-enhanced tobramycin uptake and lethality (Fig. 4C and D). Notably, NaN_3_, an inhibitor of electron transport, exhibited the same inhibitory effect as FCCP and CCCP. Nevertheless, DNP, a widely used uncoupler for PMF generation (41), exhibited little effect on glycerol-enhanced tobramycin uptake and much less inhibitory effect on lethality than CCCP/FCCP/NaN_3_. We further provided evidence, by flow cytometric analysis using fluorescence probe 3,3′-diethyloxacarbocyanine iodide, that rapid glycerol treatment greatly increased the PMF of stationary-phase *S. aureus* cells, and the presence of CCCP largely abolished such PMF enhancement (Fig. 4E). Together, these observations indicate that glycerol-induced tobramycin potentiation is achieved via PMF-dependent enhancement of tobramycin uptake.

### ARTP-based genome-wide mutagenesis screen identifies key genes essential for glycerol-induced aminoglycoside potentiation

**Fig 5:**
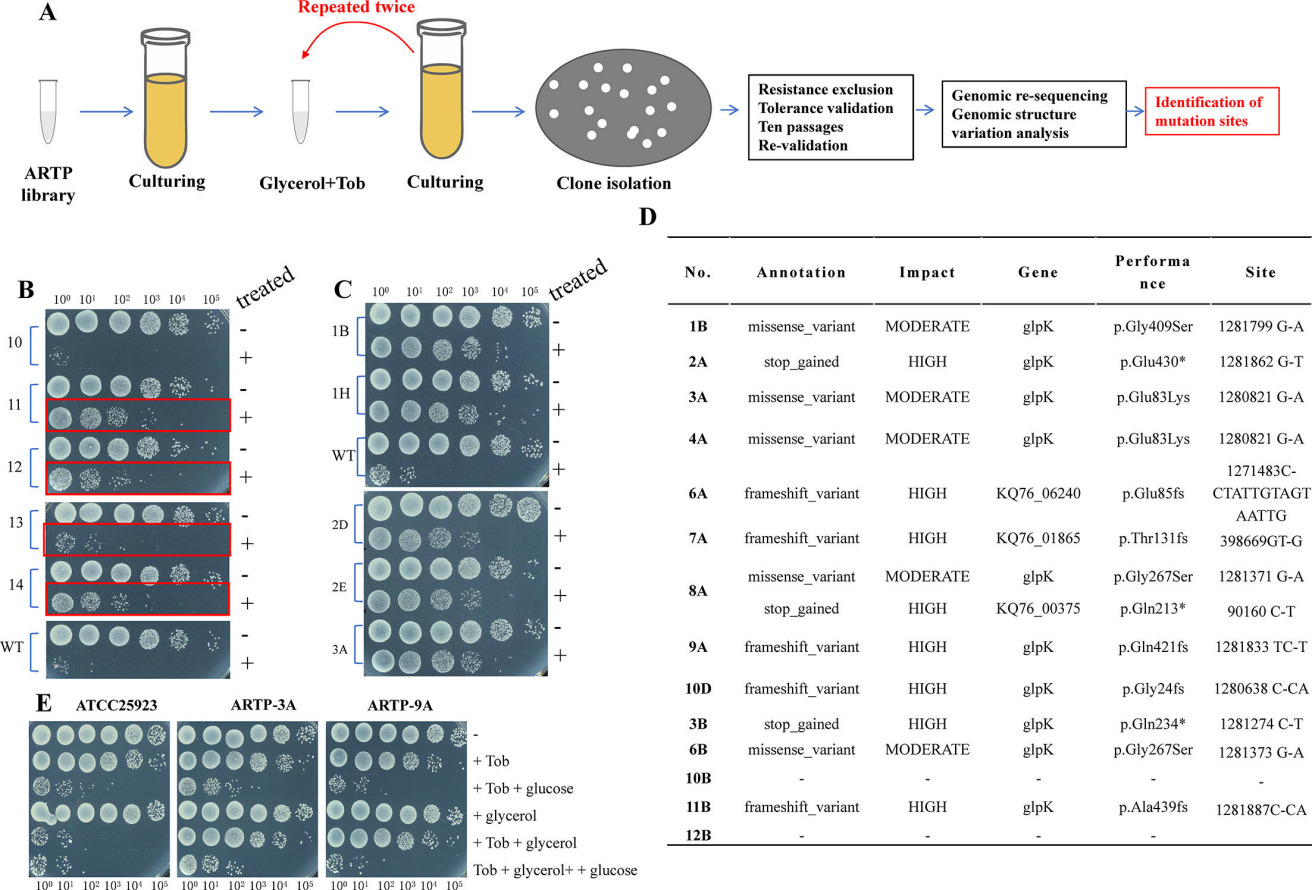
ARTP-based genome-wide screen uncovers the genetic basis of glycerol-induced tobramycin potentiation in *S. aureus.* (**A**) Schematic illustration for tolerant clone isolation and mutation site identification using *S. aureus* ARTP library (see Materials and Methods section for details). (**B**) Survival of *S. aureus* sub-libraries (after three cycles of treatment and culturing) following the combined treatment with 50 µg/mL tobramycin plus 0.3 M glycerol. (**C**) Survival of *S. aureus* tolerant clones following the combined treatment. (**D**) Mutation sites in *S. aureus* tolerant clones. (**E**) Survival of typical *S. aureus* tolerant clones (ARTP-3A and ARTP-9A, refer to panel **D**) and wild-type cells following the combined treatment with 50 µg/mL tobramycin plus 0.3 M glycerol or glucose.

We next tried to uncover the genetic basis of glycerol-induced aminoglycoside potentiation in *S. aureus*. Here, ARTP, a powerful mutagenesis tool widely used in industrial microbiology (42, 43), was explored to generate a genome-wide random mutation library of *S. aureus* (Fig. S8A), which facilitated the enrichment of specific clones that are tolerant to the tobramycin-glycerol combined treatment and then allowed us to identify the key genes via genomic re-sequencing and analysis (as diagrammed in Fig. 5A). After three rounds of enrichment (Fig. S8B to D; Fig. 5B), 8 of 14 ARTP sub-libraries exhibited certain tolerance to the combined treatment (Fig. 5B; Fig. S8D). Around 10 clones from each sub-library were picked up, and those tobramycin-resistant clones with higher MIC than the wild-type strain were excluded (Fig. S9A). The remaining clones were examined with respect to their tolerance to the combined treatment (Fig. S9B) and were confirmed to maintain such tolerance after 10 passages (Fig. 5C and Fig. S9C), which thus generated genetically stable mutants with a tolerant phenotype. Finally, a total of 14 representative tolerant clones plus the wild-type strain (*S. aureus* ATCC 25923) were subjected to genomic re-sequencing followed by structural variation analysis, and mutation sites for each clone were identified and summarized in Fig. 5D.

Several points could be drawn from Fig. 5D. First of all, among the 14 tolerant clones, there are 9 clones that each carried a unique mutation (missense, frameshift, or stop) exclusively in the *glpK* gene, which encodes glycerol kinase catalyzing the phosphorylation of glycerol to yield sn-glycerol 3-phosphate, a chemical intermediate in glycolysis and lipid metabolism (refer to Fig. S10A). Second, clone 8A carries two mutation sites, one in the *glpk* gene and the other in gene KQ76_00375, encoding dimethylallyl adenosine tRNA methylthiotransferase. Third, clones 6A and 7A carry mutation sites in genes KQ76_06240, encoding acetoin reductase, and KQ76_01865, encoding growth inhibitor pemk, respectively. Lastly, clones 10B and 12B appear not to carry mutations, possibly due to problems with genomic re-sequencing.

We envisioned that the functional loss of mutations in *glpK* may account for the acquired tolerance phenotype of the mutated clones, which is further supported by the following observations. First, these clones were also tolerant to the combined treatment with other types of aminoglycosides (gentamicin, streptomycin, or kanamycin) plus glycerol (Fig. S10B). Second, genomic re-sequencing analysis of an additional 14 tolerant clones reveals that they also exclusively carry missense, frameshift, or stop mutations in the *glpK* gene (Fig. S10C). Third, typical clones ARTP-3A and ARTP-9A, though being tolerant to tobramycin plus glycerol, were still sensitive to tobramycin plus glucose, similarly to the wild-type strain (Fig. 5E). Consistently, these two clones were also tolerant to long-time treatment with tobramycin plus glycerol but still sensitive to that with tobramycin plus glucose when drug combinations were directly added into stationary-phase cell cultures (Fig. S10D). We envision that these two clones, though being unable to catabolize glycerol because of deleterious mutations in the *glpk* gene, are still able to catabolize glucose and thus are sensitive to the combined treatment with tobramycin plus glucose. Together, these observations indicate that *glpK* is vital for glycerol-induced aminoglycoside potentiation against *S. aureus*.

### GlpK mediates glycerol-induced tobramycin potentiation against *S. aureus* by boosting energy metabolism and enhancing PMF-dependent antibiotic uptake

**Fig 6:**
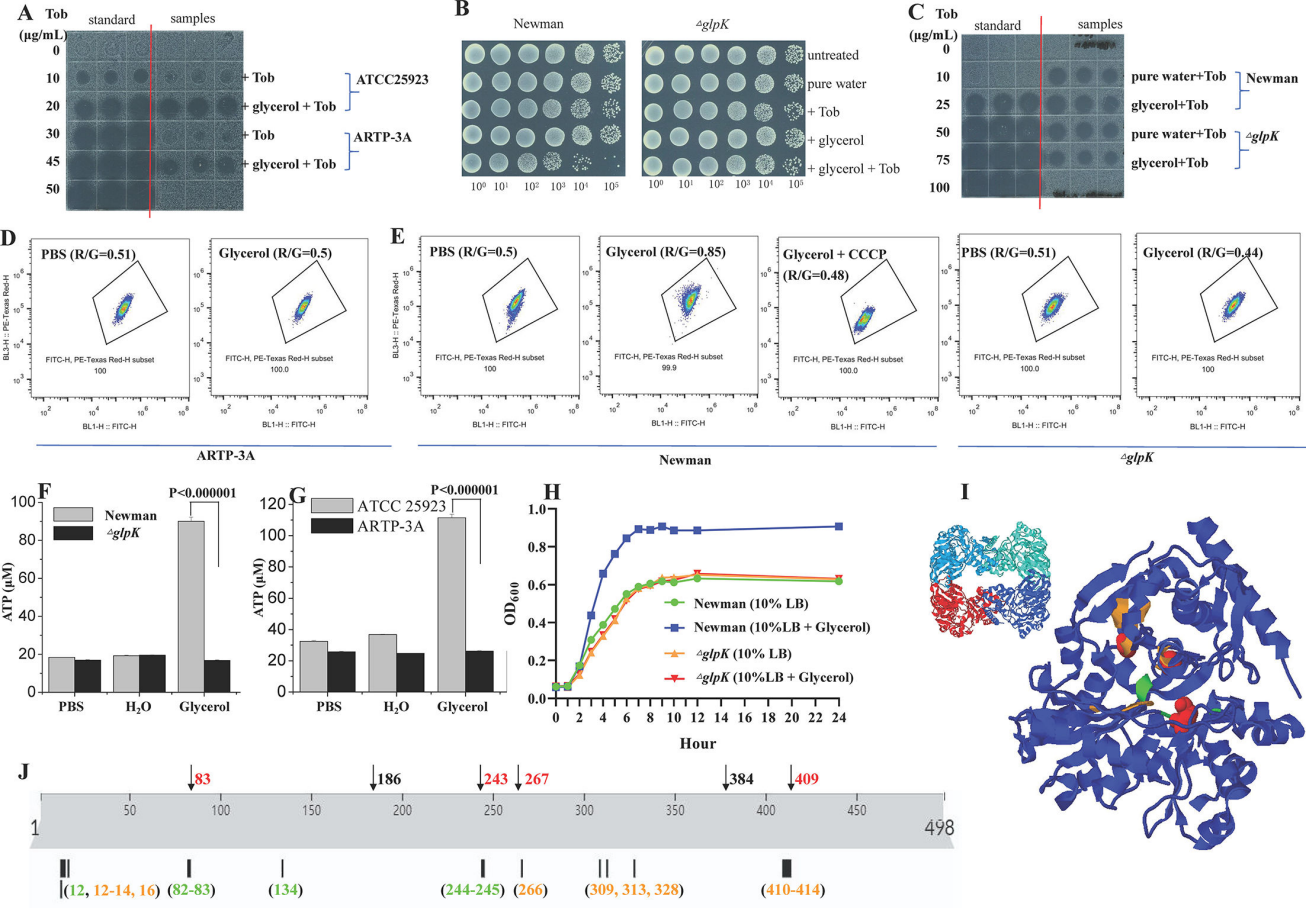
GlpK is essential for glycerol-enhanced tobramycin uptake and lethality via boosting glycerol-initiated energy metabolism. (**A**) Inhibition of *E. coli* cell growth on LB agar dishes by tobramycin extracted from stationary-phase *S. aureus* ATCC 25923 wild-type and ARTP-3A mutant cells, which were pretreated with 100 µg/mL tobramycin dissolved in pure water or 0.3 M glycerol. (**B**) Survival of *S. aureus* Newman wild-type and Δ*glpK* mutant cells following the combined treatment with 50 µg/mL tobramycin plus 0.3 M glycerol. (**C**) Inhibition of *E. coli* cell growth on LB agar dishes by tobramycin extracted from stationary-phase *S. aureus* Newman wild-type and Δ*glpK* mutant cells, which were pretreated with 100 µg/mL tobramycin dissolved in pure water or 0.3 M glycerol. (**D, E**) Results of a flow cytometric analysis of stationary-phase *S. aureus* ARTP-3A mutant cells (panel **D**) as well as Newman wild-type and Δ*glpK* mutant cells (panel E) after treatment with indicated buffers (phosphate-buffered saline [PBS] or glycerol), similarly to Fig. 4E. (**F, G**) ATP levels of indicated *S. aureus* cells after glycerol treatment. (**H**) Growth curves of Newman wild-type and Δ*glpK* mutant cells as cultured in 10% LB medium plus 0.3 M glycerol. (**I**) 3-D structure of GlpK (PDB ID: 3g25). Left: tetrameric structure of GlpK; right part: monomeric structure of GlpK showing its glycerol- and ATP-binding sites (colored in green and orange, respectively) and missense variation sites from our tolerant clones (colored in red). (**J**) Overlapping of missense variation sites of GlpK (colored in red on the top) with its ATP- and glycerol-binding sites (colored in green and orange on the bottom, respectively).

We next investigated how *glpK* contributes to glycerol-induced aminoglycoside potentiation against *S. aureus.* First, we showed that the tobramycin uptake of clone ARTP-3A, unlike the wild-type strain, could not be enhanced by glycerol (Fig. 6A). Second, we constructed a *glpK* deletion mutant in the *S. aureus* Newman strain (Note: gene deletion of *glpK* was not successfully achieved in the ATCC 25923 strain due to the failure of plasmid transformation into this strain by electroporation), and found that the Δ*glpK* mutant was fully tolerant to the tobramycin-glycerol combined treatment, whereas the Newman wild-type strain could be killed by around two orders of magnitude (Fig. 6B). Consistently, glycerol could increase the tobramycin uptake of Newman wild-type cells but not that of Δ*glpK* cells (Fig. 6C). Similarly, a comparative study between the *S. aureus* RN4220 strain and the corresponding Δ*glpK* mutant also revealed that *glpK* was essential for both glycerol-enhanced tobramycin lethality and uptake (Fig. S11A and B, respectively).

Given that glycerol-induced tobramycin potentiation against *S. aureus* ATCC 25923 cells relies on the glycerol-enhanced PMF that subsequently enhances tobramycin uptake (Fig. 4), we measured the effect of *glpK* mutation or deletion on PMF. Flow cytometric analysis revealed that rapid glycerol treatment hardly increased the PMF of clone ARTP-3A (Fig. 6D), in contrast to glycerol-induced enhancement of PMF in the wild-type strain (Fig. 4E). Consistently, glycerol increased the PMF of Newman wild-type cells but not Δ*glpK* cells, and CCCP co-treatment could abolish such increase in the PMF of wild-type cells (Fig. 6E).

In addition, given that the production of ATP relies on PMF and the driving force of aminoglycoside uptake is likely ATP (44), we examined the effect of *glpK* on ATP level. Luciferase assay revealed that rapid glycerol treatment markedly elevated ATP levels of both Newman and ATCC 25923 wild-type cells but had little effect on Δ*glpK* and clone ARTP-3A mutant cells (Fig. 6F and G). In addition, the supplementation of glycerol in the LB medium for cell culturing could promote cell growth and increase the biomass of Newman wild-type cells but had little effect on Δ*glpK* cells (Fig. 6H). Together, these observations suggest that GlpK-catalyzed conversion of glycerol to sn-glycerol 3-phosphate boosts the energy metabolism of *S. aureus* cells (PMF and ATP levels), which, in turn, enhances the bacterial uptake and bactericidal action of aminoglycoside antibiotics. Nevertheless, such glycerol-enhanced bactericidal action of tobramycin did not induce apparent cell morphology damage as revealed by scanning electron microscopic analysis (Fig. S11C). In addition, we did not detect any alterations on the mRNA levels of *glpF* and *glpK* upon glycerol treatment (Fig. S11D), indicating that *S. aureus* cells respond to the addition of glycerol by using the existing GlpK protein to catalyze glycerol phosphorylation.

How do those mutations in *glpK* lead to the loss of its function? We looked into the missense mutations and found that they are partially overlapped or very close in proximity to the ATP- and glycerol-binding sites of GlpK in its 3-D structure (Fig. 6I). Along the primary sequence of GlpK, the missense mutation sites from several ARTP-mutated clones (colored in red on the top; Fig. 6J) are apparently located within the ATP- and glycerol-binding sites of GlpK (colored in green and orange on the bottom, respectively). Hence, it appears that these missense mutations most likely disrupt the catalytic activity of GlpK and thus render mutated clones tolerant to the tobramycin-glycerol combined treatment.

## DISCUSSION

### Glycerol-induced aminoglycoside potentiation is achieved by rapidly boosting GlpK-mediated energy metabolism and enhancing antibiotic uptake

Bacterial metabolism is crucial for antibiotic efficacy (11), and therefore modulation of bacterial metabolism is accordingly able to alter antibiotic efficacy. Such a metabolic reprogramming-based antibiotic potentiation strategy has been demonstrated with various types of metabolites, including glucose, mannitol, formate, fumarate, glycerol, alanine, cis-2-decenoic acid, N-acetyl-D-glucosamine, and metformin, toward aminoglycosides, fluoroquinolones, β-lactams, and/or tetracyclines (8, 16–18, 23–27, 34). These metabolites undergo extensive catabolism in bacteria during co-treatment for a couple of hours, which, in turn, usually alters the metabolic status of bacteria. In particular, aminoglycosides are of interest for efficacy enhancement, as their bacterial uptake and downstream lethal actions are both linked to cellular respiration (27, 45).

**Fig 7:**
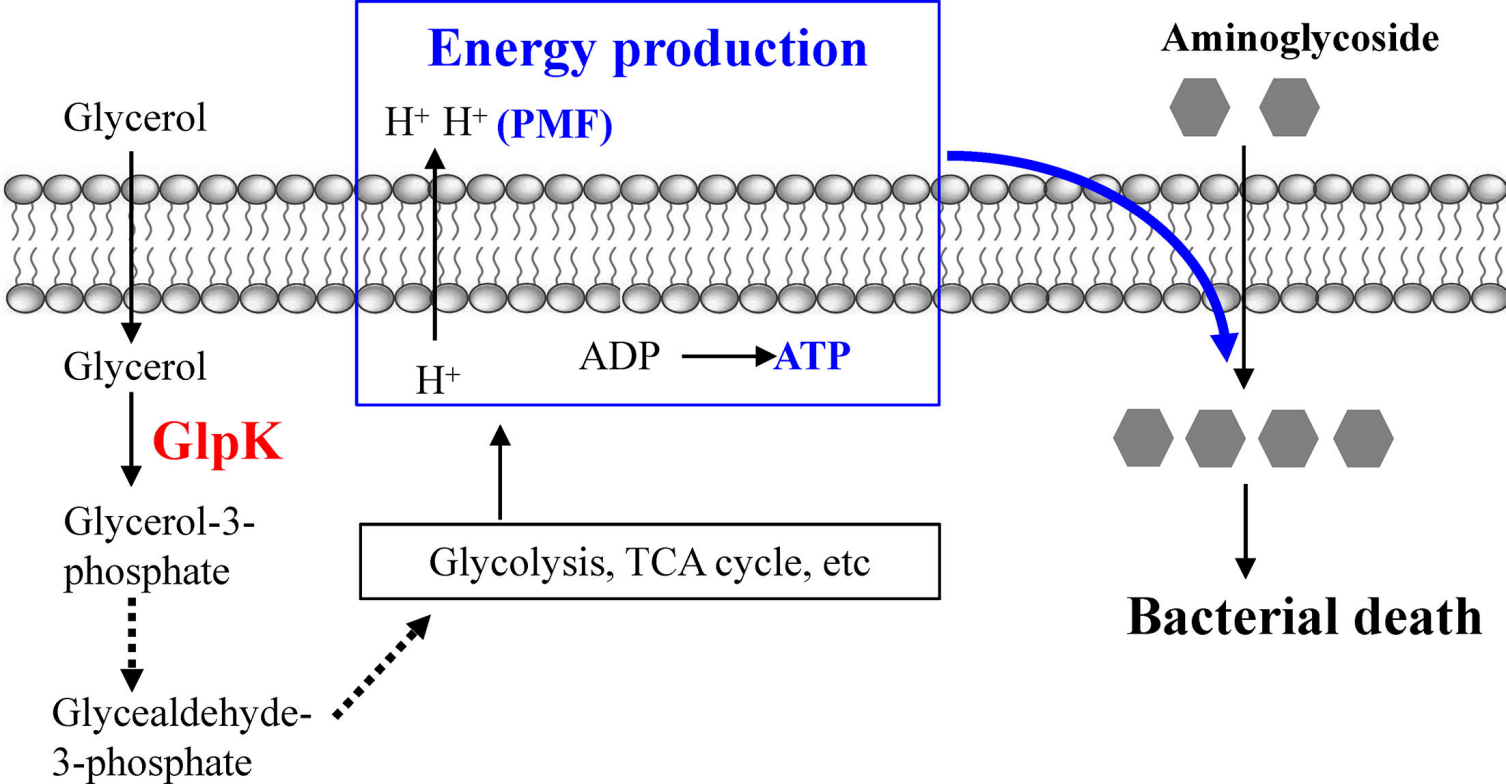
Mechanistic illustration for glycerol-induced aminoglycoside potentiation. The key to potentiating aminoglycosides by glycerol is to facilitate aminoglycosides to traverse the cytoplasmic membrane of bacteria cells under the driving force PMF. Apparently, glycerol catabolism, as initiated by the first key enzyme GlpK, would boost the energy metabolism of whole cells, eventually elevating PMF and ATP levels and thus enhancing aminoglycoside uptake and lethality.

The significance of our aminoglycoside-glycerol combination approach lies in the very short duration for co-treatment, which can be as short as 1 min or even 10 seconds (Fig. 1E), somehow similar to physical interference approaches such as hypoionic shock, rapid freezing, and heat shock, as we developed recently (28–30, 46). Despite huge differences, the nature of both long-term and rapid aminoglycoside-glycerol co-treatments may be the same, i.e., both of which seem to be achieved via boosting or reprogramming bacterial energy metabolism (e.g., glycolysis and tricarboxylic acid [TCA] cycle) that subsequently enhances aminoglycoside uptake in a PMF-dependent manner (as diagrammed in Fig. 7). Given that bacterial persisters are usually metabolically repressed (13), such metabolite-stimulated energy metabolism thus enables aminoglycosides to effectively eradicate persisters as reported earlier (17) or here (Fig. 2A and B). As such, *glpK,* essential for glycerol catabolism, is somehow unsurprisingly identified as a dominant gene for glycerol-mediated aminoglycoside potentiation. Consequently, deletion or deleterious mutations of *glpK* abolished glycerol-boosted energy metabolism (Fig. 6D through F) and thus tobramycin uptake (Fig. 6A and C).

Our approach is apparently distinct from rhamnolipid-based strategy, wherein the enhanced aminoglycoside uptake does not involve the metabolism of rhamnolipids (47). Notably, our approach is largely independent of the presence of excessive NaCl, thus behaving distinctively from 5-methylindole- or *n*-butanol-induced aminoglycoside potentiation (31, 32), two approaches we developed earlier that are largely inactive in the presence of NaCl. Together, our approach represents a unique scenario for aminoglycoside potentiation with respect to both action and mechanism.

### Therapeutic potential of glycerol-induced aminoglycoside potentiation against bacterial infection

Aminoglycosides have been widely used since the discovery of streptomycin in the 1940s (48). However, their clinical application has declined significantly due to dose-limiting toxicity and the rising prevalence of antibiotic resistance (49). Overcoming resistance and reducing toxicity are therefore crucial for maximizing the therapeutic potential of this important class of bactericidal antibiotics, particularly in the context of carbapenem-resistant bacterial infections. Our approach holds clinical significance for the following reasons: (i) it enhances the bactericidal activity of aminoglycosides by more than two orders of magnitude; (ii) it effectively kills biofilms and persister cells, which are key contributors to chronic and recalcitrant infections; and (iii) the combined treatment requires only a short exposure time (a few minutes), which may help limit systemic toxicity—especially if the drug is promptly removed after treatment. Furthermore, topical applications of aminoglycosides (e.g., tobramycin-containing ophthalmic solutions for bacterial keratitis) may benefit more from short-term co-treatment with glycerol than from prolonged exposure, given the potential for rapid diffusion of both the drug and the potentiator.

Although various types of antibiotic adjuvants have been discovered and even observed to be effective in curing infections in animal models (8, 16, 50), only β-lactamase inhibitors have been successfully applied in the clinic so far. In this regard, of particular interest in our study is that we demonstrate, as a proof of concept, the much-improved therapeutic efficacy of tobramycin against *S. aureus* infection *in vivo* via merely replacing NaCl with glycerol. Given the GRAS characteristic of glycerol, these advantages make this combined treatment very attractive for clinical trials.

### Potential of ARTP-based genome-wide mutagenesis for bacterial genetic research

Genome-wide screen is a powerful tool for delineating the genetic basis of any biological phenotype in both prokaryotic and eukaryotic cells. For instance, gene deletion and expression libraries (51, 52), transposon libraries (53–55), as well as CRISPRi libraries (56) have been widely used in identifying the genes involved in the antibiotic resistance and tolerance of bacterial pathogens. In contrast to these libraries, ARTP-based genome-wide mutagenesis library usually contains millions of clones, and each clone carries one or a few base-pair alterations in the genome of bacteria, which result in missense, frameshift, stop-gained, or synonymous mutations in one or a few genes (43). Apparently, this approach has two advantages: first, it nicely mimics point mutations, instead of the loss of a whole gene, that ubiquitously and spontaneously take place in all living organisms; second, combined mutations in multiple genes, rather than an alteration in one gene, may generate synthetic phenotype.

ARTP has been widely applied in industrial microbiology to generate mutants with improved yield of specific bio-products (42) and was recently explored for biofilm research (57), but it has not been reported for antibiotic resistance research so far. Here, we demonstrate its potential in identifying the critical genes for glycerol-induced aminoglycoside potentiation. Generally speaking, this strategy can be easily explored as a general genome-wide screen approach to isolate bacterial mutants that are resistant or tolerant to specific antibiotics and disinfectants, to detrimental abiotic stresses such as heat shock, low pH, osmotic shock, and UV radiation, as well as to other biological attacks from bacteriophages or the immune system of the host. Some of these potential applications are being carried out in our laboratory, which may guide us to uncover untapped genes essential for cell survival under specific stressful conditions.

## MATERIALS AND METHODS

### Bacterial strains, plasmids, and reagents

Bacterial strains used in this study include *S. aureus,* MRSA, *Staphylococcus epidermidis*, *Enterococcus faecalis*, *Micrococcus luteus*, *Pseudomonas aeruginosa*, *E. coli*, *Shigella flexneri, Acinetobacter baumannii, Klebsiella pneumoniae,* and *Salmonella typhimurium* (Table S1). Clinical isolates of MRSA and methicillin-sensitive *S. aureus* are presented in Tables S2 and S3, respectively. Plasmids for gene knockout and expression are shown in Table S4, with the primers for plasmid preparation presented in Table S5. Chemical reagents are of analytical purity.

### Combined treatment with aminoglycoside and glycerol

Overnight cell cultures were diluted 1:200 in LB medium and cultured for 24 h to prepare stationary-phase cells, of which 30 μL of culture was centrifuged (12,000 *g*, 1 min) in Eppendorf tubes and the supernatant was fully removed. Cell pellets were thoroughly re-suspended by pipetting 10 times in 100 μL working solution containing aminoglycoside and glycerol (or other carbon source), and further incubated at room temperature for varying lengths of time (usually for 3 min). Cell suspensions were washed twice with phosphate-buffered saline (PBS; 0.27 g/L KH_2_PO_4_, 1.42 g/L Na_2_HPO_4_, 8 g/L NaCl, 0.2 g/L KCl, pH 7.4) by centrifugation (12,000 *g*, 30 seconds), and then 4 µL of 10-fold serially diluted cell suspension was spot-plated onto LB agar dishes for survival assay. In addition, overnight cell cultures of *S. aureus* were diluted 1:1,000 in LB medium and cultured for 6 h to prepare exponential-phase cells before 100 µL of cell culture was subjected to the same treatment.

### Preparation and eradication of *S. aureus* persister cells

*S. aureus* exponential-phase cells (OD_600_ ≈ 0.5) were pretreated with 25 μg/mL tobramycin for 3 h to generate spontaneous persisters, which were then subjected to a 3 min combined treatment with 25 μg/mL tobramycin dissolved in pure water or 0.3 M glycerol solution. Starvation*-*induced persisters were prepared as previously reported (37): stationary-phase *S. aureus* cells, as cultured in MHB medium, were transferred into YNB medium and starved for 5 h before being subjected to the combined treatment.

### Preparation and eradication of *S. aureus* biofilms *in vitro*

Overnight *S. aureus* cell cultures were diluted 1:500 into 2 mL of fresh LB medium containing six polyethylene (PE) tubes in a flask and agitated (37°C, 220 rpm) for 72 h, with LB medium being changed every 24 h. PE tubes with *S. aureus* biofilms were washed twice with PBS, and then treated with 50 μg/mL tobramycin plus 0.3 M glycerol for 5 min. After washing twice with PBS, biofilms were removed from the PE tubes by sonication and then spotted on LB agar dishes for cell survival assay.

### Biofilm infection in mice

A 1 cm × 1 cm skin wound was generated in mice as described above, and then was seeded with around 1 × 10^7^ colony-forming units (CFU) stationary-phase *S. aureus* cells before sealing with petrolatum and wrapping with medical gauze. After 48 h of housing, the biofilms formed in the skin wound were subjected to a 10 min combined treatment with 200 μg/mL tobramycin dissolved in 0.9% NaCl solution or 0.3 M glycerol solution before cell survival assay as described above.

### Genome-wide screen of tolerant clones using *S. aureus* ARTP library

ARTP mutagenesis was performed according to the manufacturer’s instructions such that the plasma radiation-induced cell death ratio for exponential-phase *S. aureus* ATCC 25923 cells was around 90%, with a total of 14 ARTP sub-libraries being generated and stored in a freezer. Each sub-library was recovered in LB medium for 2 h and subjected to a 3 min combined treatment with 50 μg/mL tobramycin plus 0.3 M glycerol. The surviving cells were re-cultured in LB medium and subjected to the combined treatment again. After three rounds of treatment and culturing, the sub-libraries possessing tolerance to the combined treatment were subjected to tolerant clone selection and validation, including the exclusion of tobramycin-resistant mutants and 10-passage verification. Typical tolerant clones from each sub-library were subjected to next-generation sequencing on the Illumina platform by Novogene Co., Ltd. (Beijing, China). Genomic structural variation analysis was conducted based on the reference genome of *S. aureus* ATCC 25923 using such tools as Trimmomatic (version 0.39), FastUniq (version 1.1), SPAdes (version 3.13.0), Samtools (version 1.1), Picard (version 2.27.1), Bowtie2 (version 2.2.4), GATK (version 4.2.6.1), and SnpEff (version 5.0e), such that mutation sites of each tolerant clone were identified.

### Statistics

CFU from LB agar dishes during the cell survival assay were counted, and cell density was calculated according to the dilution fold and volume of cell droplet. Statistical analysis was performed in the MicroOrigin software using the analysis of variance algorithm at a significance level of 0.05.

Other methods, including ATP level assay, PMF measurement, skin infection in mice, gene knockout and complementary expression, preparation of competent cells, and plasmid transformation, are presented in the supporting information file.

## Data Availability

The data that supports the findings of this study are available in the Supplemental material of this article, and the re-sequenced genome of mutants are deposited as BioProject ID PRJNA1332149.

## SUPPLEMENTAL MATERIAL

Supplemental material aac.00938-25-s0001.pdf Tables S1 to S5; Fig. S1 to S11; Supplemental methods.

ASM does not own the copyrights to Supplemental Material that may be linked to, or accessed through, an article. The authors have granted ASM a non-exclusive, world-wide license to publish the Supplemental Material files. Please contact the corresponding author directly for reuse.
