# Supplementary material for: Genome-wide screen reveals glycerol-induced aminoglycoside potentiation against Staphylococcus aureus via boosting GlpK-initiated energy metabolism
Source: Antimicrob Agents Chemother. 2025 Oct 23;69(12):e00938-25. doi: 10.1128/aac.00938-25 (PMC12691589; doi:10.1128/aac.00938-25)
Supplement: Supplemental material — Tables S1 to S5; Fig. S1 to S11; Supplemental methods. [file aac.00938-25-s0001.pdf]

Supporting Information

Genome-wide screen reveals glycerol-induced aminoglycoside potentiation against *Staphylococcus aureus* via boosting GlpK-initiated energy metabolism

Yaqin Xing [1, #], Mengmeng Bian [1, 2, #], Xuebing Huang [1, 2, #], Boyan Lv [1, #], Zhijie Huang [1], Xianzhang Jiang [2], Weiya Huang [1], Huping Xue [3], Hangyu Zhao [1], and Xinmiao Fu [1, 2, *]

[1] Provincial University Key Laboratory of Cellular Stress Response and Metabolic Regulation, College of Life Sciences, Fujian Normal University, Fuzhou City, Fujian Province 350117, China

[2] Institute of Precision Medicine, Fujian Provincial Hospital, Fuzhou University Affiliated Provincial Hospital, Fuzhou City, Fujian Province 350001 China

[3] Department of Animal Science and Technology, University of Northwest A&F, Yangling, Shaanxi, China

[#] These authors contributed to this work equally.

[*] To whom correspondence should be addressed: Professor Xinmiao Fu (xmfu@fjnu.edu.cn)

Methods

Intracellular ATP level assay

A luciferase-based kit (BacTiter-Glo™ Microbial Cell Viability Assay, Promega Corporation, USA; Cat.# G8093) was used to measure ATP level according to the manufacturer's instruction. Briefly, *S. aureus* stationary-phase cells, with or without pretreatment of sodium arsenate, CCCP, FCCP, NaN_3 or DNP for one hour, was directly mixed with the working solution at equal volumes, incubated for 5 min and transferred into a 96-well plate before light recording on a FLUOstar Omega Microplate Reader using the Luminometer method.

Proton motive force assay

A flow cytometry-based assay was applied to measure PMF by using fluorescence probe 3,3'-Diethyloxacarbocyanine Iodide (DiOC2(3)); purchased from MaoKang Biotechnology, Inc., Shanghai, China) according to the manufacturer's instruction. Briefly, *E. coli* persisters, with or without CCCP (or *n*-butanol) pretreatment as described above, were diluted into PBS to a cell density of 10^6 cells/mL and incubated with DiOC2(3) (at a final concentration of 30 μM) at room temperature for 30 min. Cells were subjected to flow cytometry analysis on FACSymphony™A5 (BD Biosciences) with an excitation at 488 nm and emission at both red and green channels.

Skin wound infection in mice

We applied a skin acute wound model as previously described (28). In brief, 8-week-old ICR male mice (around 28 g) were randomly divided into three groups for surgery experiments (Group A: treatment with 0.9% NaCl solution; Group B: treatment with 50 μg/mL tobramycin in 0.9% NaCl solution; Group C: treatment with 50 μg/mL tobramycin in 0.3 M glycerol solution; n=6). Mice were anesthetized by intraperitoneal injection of 4% chloral hydrate, barbered on the right back and sterilized, and then a 1cm×1cm whole skin section was removed to make an acute skin wound. Afterwards, 5 μL of tenfold-concentrated stationary-phase *S. aureus* cells (approximately $5x10^7$ cfu) in NaCl solution were seeded on the wound and fully absorbed before adding 120 μL working solution and further incubating for 5 min. Residue working solution was removed by absorbing with medical cotton. After full drying, mice were bandaged firmly with medical gauze and housed over-night. The whole scab on the wound site was removed and homogenized, with the lysate being

spot-plated on LB agar dishes for bacterial survival assay.

Knockout and complementary expression of glpK in S. aureus

Bacterial strains, plasmids and primers for gene knockout and expression in *S. aureus* Newman and RN4220 strains are presented in **Table S1**, **S4** and **S5**, respectively. Briefly, knockout of *glpK* was conducted using pKZ2 plasmid (GenBank ID: KY615709) and 750 bp sequences up- and down-stream of the *glpK* gene in Newman strain were cloned into pKZ2 for DNA homologous recombination via PCR amplification and Easy cloning one step (TransGen Biotech, Beijing). *E. coli* DH5α strain was used for cloning, and *E. coli* IM08B strain was used for plasmid methylation that enables the plasmid to be degradation-resistant in competent *S. aureus* cells after transforming via electroporation. Positive clones growing on chloramphenicol-containing BHI agar dishes were isolated, and DNA homologous recombination was achieved by 1:1000 diluting into BHI medium and culturing at 43°C for up to 48 h. The remaining pKZ2 plasmid was removed by consecutive passage or 0.5 μg/mL anhydrotetracycline-mediated negative screen, and gene knockout was confirmed by PCR amplification. In addition, *glpK* gene was cloned into pSE1 by conventional PCR amplification and ligation and its expression was under the control of the promoter of *mecA* gene from *S. aureus* cells (5341..5439bp). The pSE1-*glpK* plasmid, as purified from *E. coli* DH5α, was modified in *E. coli* IM08B and then transformed into *S. aureus* Newman and RN4220 *△glpK* strains for complementary expression of GlpK.

Preparation of competent S. aureus cells and electroporation-mediated plasmid transformation

RN4220 cells (or other *S. aureus* strains) were picked up from -80°C freezer and restored over-night in BHI medium at 37°C. Cells were sub-cultured in BHI medium after 1:100 dilution, collected at OD_{600}=0.8, washed twice with pure water and then re-suspended with sterilized 10% glycerol. After centrifugation, cells were re-suspended with sterilized 10% glycerol, sub-packaged in EP tubes at 66 μL and frozen in nitrogen for 10 sec before stored in -80°C freezer. For plasmid transformation, competent cells in EP tube were picked up from -80°C freezer, mixed with 10 μL plasmid (around 10 μg) and incubated on ice for 20 min. The mixture was transferred into sample chamber and electroporation was conducted at the following parameters: 2.5 KV, 200 ohm, 50 μF and 5 ms. Cells were quickly mixed with 1 mL BHI medium and recovered at 37°C for 2 h before plating on 10 μg/mL chloramphenicol-containing LB agar dish for positive clone isolation.

mRNA assay by RT-qPCR

Stationary-phase *S. aureus* cells (ATCC25923) were treated with pure water or 0.3 M glycerol for 3 minutes and then subjected to mRNA extraction using FastPure Cell/Tissue Total RNA Isolation Kit V2 (Vazyme Biotech Co.,Ltd). cDNA was generated using Hifair® III 1st Strand cDNA Syntheis SuperMix for qPCR (YEASEN

Inc) and quantified using Hieff UNICON® Advanced qPCR SYBR Master Mix (gDNA digester plus) from YEASEN, Inc., with gyrB being as the internal control.

Results

Table S1 Bacterial strains used in this study

No	Bacterial strains	Origins	Characteristics
1	*Staphylococcus aureus* ATCC29523	A gift from Dr. Luhua Lai at Peking University	G+
2	MRSA ATCC43300	Purchased from Shanghai Luwei Microbiology Technology Co., Ltd.	G+, multi-drug resistant
3	*S. aureus* Newman	A gift from Dr. Huping Xue at Northwest A&F University	G+
4	*S. aureus* RN4220	A gift from Dr. Huping Xue at Northwest A&F University	G+
5	*Staphylococcus epidermidis* CMCC26069	Purchased from Hangzhou Binhe Microorganism Reagent Co., Ltd.	G+, streptomycin -resistant
6	*Enterococcus faecalis* ATCC29212	Purchased from Hangzhou Binhe Microorganism Reagent Co., Ltd.	G+, multi-drug resistant
7	*Micrococcus luteus* CMCC28001	A gift from Dr. Qingeng Huang at Fujian Normal University	G+,tobramycin-and kanamycin-resistant
8	*Shigella flexneri* 24T7T	A gift from Prof. Xiaoyun Liu at Peking University	G+, streptomycin -resistant
7	*Escherichia coli* BW25113	Purchased from the Nara Institute of Science and Technology(Ikoma,Nara,Japan)	G-
8	*Pseudomonas aeruginosa* PA01	A gift from Dr. Zhengxian Tian at Peking University	G-,kanamycin-resistant
9	*Acinetobacter baumannii* Ab6	A gift from Prof. Xuanxian Peng at Sun Yat-Sen University	G^-, multi-drug resistant
10	*Klebsiella pneumoniae* KP-D367	The same as above	G^-, multi-drug resistant
11	*Salmonella*	A gift from Prof. Xiaoyun Liu at	G^-, Streptomycin-resistant

	typhimurium SL1344	Peking University	
12	*E.coli* DH5α	A strain stored in our laboratory	For gene cloning
13	*Ecoli* IM08B	A gift from Prof. Xue Liu at Shenzhen University	For generating plasmids that are resistant to degradation in *S. aureus* cells
14	RN4220-△*glpK*	Made in this work	The RN4220 derived strain with *glpK* being deleted
15	Newman-△*glpK*	Made in this work	The Newman derived strain with *glpK* being deleted

Table S2 Characteristics of MRSA clinical isolates

	**1**	**2**	**3**	**4**	**5**	**6**	**7**	**8**	**9**
	623520	1164569	6783332	1156650	719894	1158374	1159945	1180176	832590
Gentamicin	R	S	S	S	I	S	R	S	S
Rifampicin	S	S	S	S	S	S	R	S	S
Methicillin	R	R	R	R	R	R	R	R	R
Inducible clindamycin	S	S	S	S	S	S	R	S	S
Ciprofloxacin	R	S	S	S	S	R	R	S	S
Moxifloxacin	R	S	S	S	S	R	R	S	S
Tetracycline	R	S	S	R	S	S	R	R	S
Teicoplanin	S	S	S	S	-	S	S	-	S
Cotrimoxazole	S	S	S	R	S	S	S	S	S
Clindamycin	R	R	S	R	R	S	R	R	R
Linezolid	S	S	S	S	S	S	S	S	S
Penicillin G	R	R	R	R	R	R	R	R	R
Erythromycin	R	R	S	R	R	S	R	R	R
Levofloxacin	R	S	S	S	S	R	R	S	S
Oxacillin	R	R	R	R	R	R	R	R	R
Tigecycline	S	S	S	S	S	S	S	S	S
Vancomycin	S	S	S	S	s	S	S	S	S
Nitrofurantoin	-	-	S	-	-	-	-	S	-
	**10**	**11**	**12**	**13**	**14**	**15**	**16**	**17**	**18**
Gentamicin	1168490	1165454	1143493	880819	1167017	1167364	1154943	1093279	1181338
Gentamicin	S	S	S	S	S	S	S	R	S
Rifampicin	S	S	S	S	S	S	S	R	S
Methicillin	R	R	R	R	R	R	R	R	R
Inducible clindamycin	S	S	S	R	S	S	S	S	R
Ciprofloxacin	S	S	S	S	S	S	S	R	S
Moxifloxacin	S	S	S	S	S	S	S	R	S
Tetracycline	S	S	S	R	S	R	S	R	S
Teicoplanin	S	-	S	S	S	S	S	S	-
Cotrimoxazole	S	S	S	S	S	S	S	S	S
Clindamycin	R	R	R	R	S	R	S	S	R
Linezolid	S	S	S	S	S	S	S	S	S
Penicillin G	R	R	R	R	R	R	R	R	R
Erythromycin	R	R	R	R	S	R	S	S	R
Levofloxacin	S	S	S	S	S	S	S	R	S
Oxacillin	R	R	R	R	R	R	R	R	R
Tigecycline	S	S	S	S	S	S	S	S	S
Vancomycin	S	S	S	S	S	S	S	S	-
Nitrofurantoin	-	-	-	S	S	S	-	-	-

Table S3 Characteristics of *S. aureus* clinical isolates

Strain No.	**1**	**2**	**3**	**4**	**5**
Antibiotics	210110897	210111330	210110881	210303453	21045914
Penicillin	R	R	R	R	R
Rifampicin	I	S	R	S	S
Ciprofloxacin	S	S	R	R	S
Levofloxacin	S	S	R	R	S
Moxifloxacin	S	S	I	R	S
Co-Trimoxazole	S	S	S	R	S
Clindamycin	S	S	S	R	S
Erythromycin	S	S	R	R	S
Linezolid	S	S	S	S	S
Vancomycin	S	S	S	S	S
Quinupristin-dalfopristin	S	S	S	S	S
Tetracycline	S	S	S	S	S
Oxacillin	R	S	R	S	S
Tigecycline	-	S	S	S	S
Gentamicin	S	S	R	R	S

Table S4 Plasmids used in this study

Name	Characteristics	Origins
pKZ2	A shuttle plasmid between *E. coli* (ampicillin resistance) and *S. aureus* (chlorampenicol resistance), knocking out gene in *S. aureus*	A gift from Prof. Huping Xue at at Northwest A&F University [2]
pSE1	A shuttle plasmid between *E. coli* (ampicillin resistance) and *S. aureus* (chlorampenicol resistance), expressing protein in *S. aureus*	A gift from Prof. Huping Xue at at Northwest A&F University
pKZ2-△*glpK*	The plasmid for knocking out *glpK* in *S. aureus*	Made in this work
pSE1-*glpK*	The plasmid for expressing GlpK in *S. aureus*	Made in this work

Table S5 Primers used in this study

name	Sequence (5'-3')	Length of target
923 *glpK*-up	GACCAAGGAACAACAAGC	1456 bp
923 *glpK*-down	CTTGTGTTGCTTCAACAGC	
pKZ2-R	CCCGATTTAAGCACACCCTTT	
pKZ2-F	AATGTCACTAACCTGCCCCGT	328 bp
KpnI-*glpK*-up	GACGTTGAGCCTCGGAACCGGTACCGATGGGGATTAGCGGTTAC	
EcoRI-*glpK*-down	ATCTCCGGCGGCCGCTCGGAATTCCTTCTAACATCATCAACCCATG	
glpK-up-Rlong	GTTAGTAGTATCTCCTTTATTAAAAAAATAATCG	1693 bp
glpK-down-Flong	TAATAAAGGAGATACTACTAACTTGTAGATTAGACTTTTGTATAAA	
923glpK validation-F	GCAGAATTCCTAGGAACTGC	
923glpK validation-R	GCTTGACCTAATGGGAATTTTG	3392 or 1895 bp
pSC1-KpnI-F	ATAAGGAGGATAATGGGTACCATGGAAAAATATATTTTATCTATAGACCAAGG	
pSC1-EcoRI-R	TGATGGATATCTGCAGAATTCTTATTCTGTTTTAAAAACTTGTGTTGCTTCAA	1497 bp
p SC1-F	TATCCTAACAGCACAAGAGC	202 bp
p SC1-R	TGTAAAACGACGGCCAGT	

Figures and Legends

Figure S1

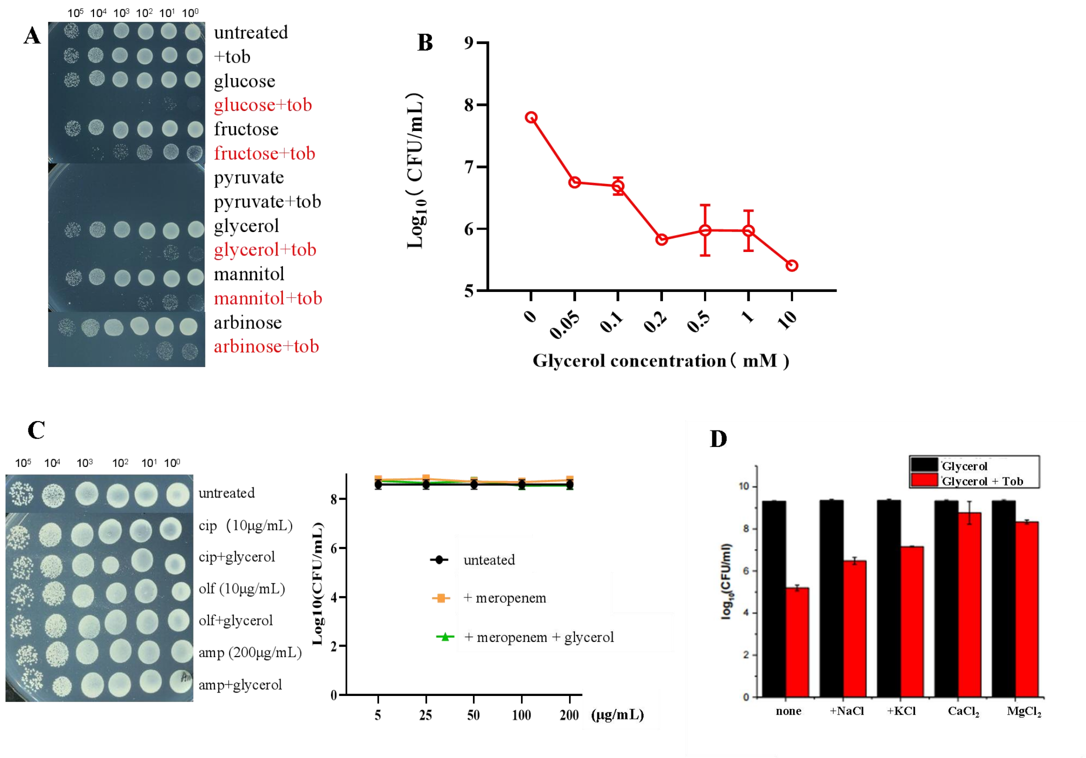

Figure S1 Characterization of glycerol-induced aminoglycoside potentiation against stationary-phase *S. aureus* cells

(**A**) Survival of stationary-phase *S. aureus* cells following a 3-min treatment with 50 μg/mL tobramycin in the presence of 0.3 M indicated carbon sources. (**B**) Quantified survival of stationary-phase *S. aureus* cells following a 3-minute treatment with 50 μg/mL tobramycin plus increasing concentrations of glycerol. (**C**) Survival of stationary-phase *S. aureus* cells following a 10-minute treatment with ciprofloxacin, ofloxacin, ampicillin and meropenem at indicated concentrations plus 0.3 M glycerol. (**D**) Survival of stationary-phase *S. aureus* cells following a 3-min treatment with 50 μg/mL tobramycin plus 0.3 M glycerol in the presence of 0.15 M NaCl or KCl, 0.1M $CaCl_2$ or $MgCl_2$.

Figure S2

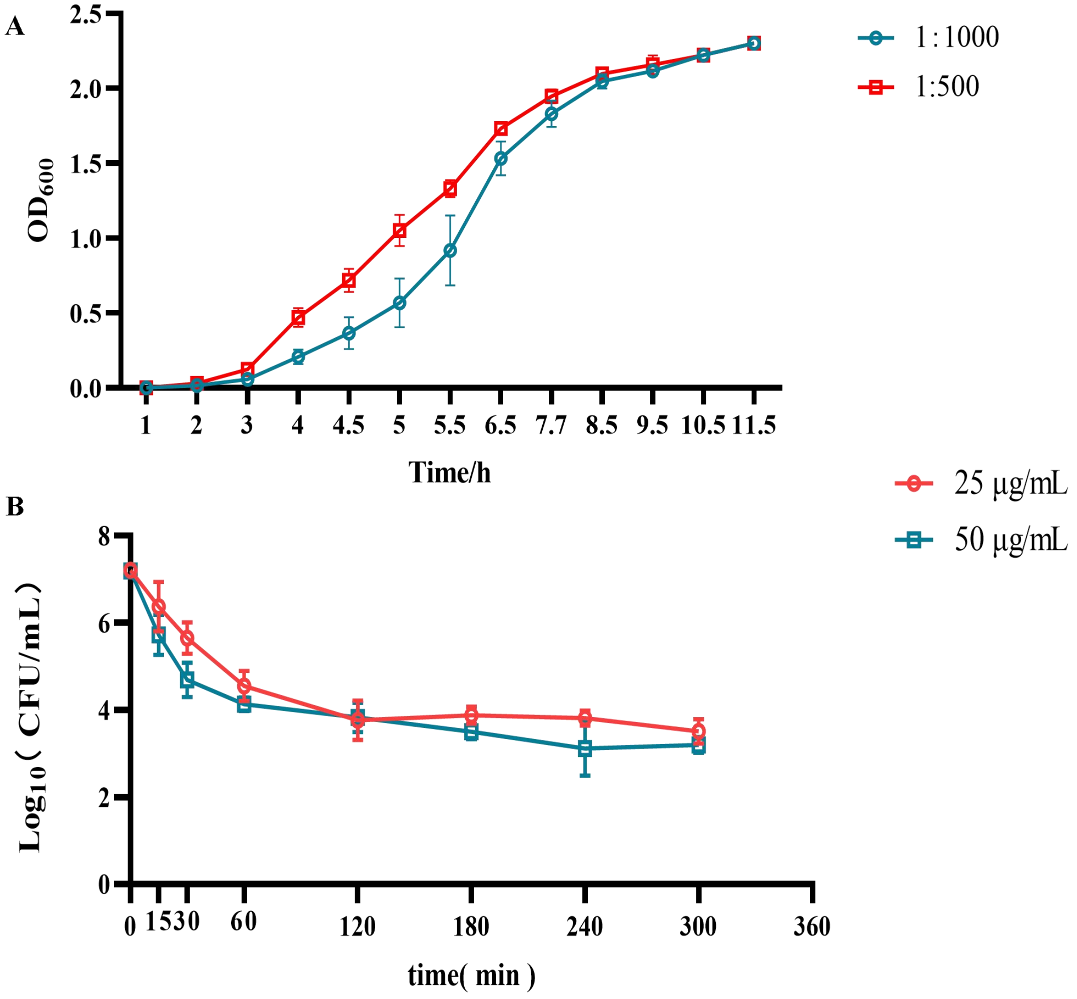

Figure S2 Growth curve of S. aureus cells and its time-killing by tobramycin

(**A**) Time-dependent OD at 600 nm of *S. aureus* cells after 1:500 or 1:1000 dilution in LB medium. (**B**) Quantified survival of *S. aureus* exponential-phase cells at OD_{600}=0.5 following a 3-minute treatment with 25 or 50 μg/mL tobramycin for varying length of time.

Figure S3 (MASA)

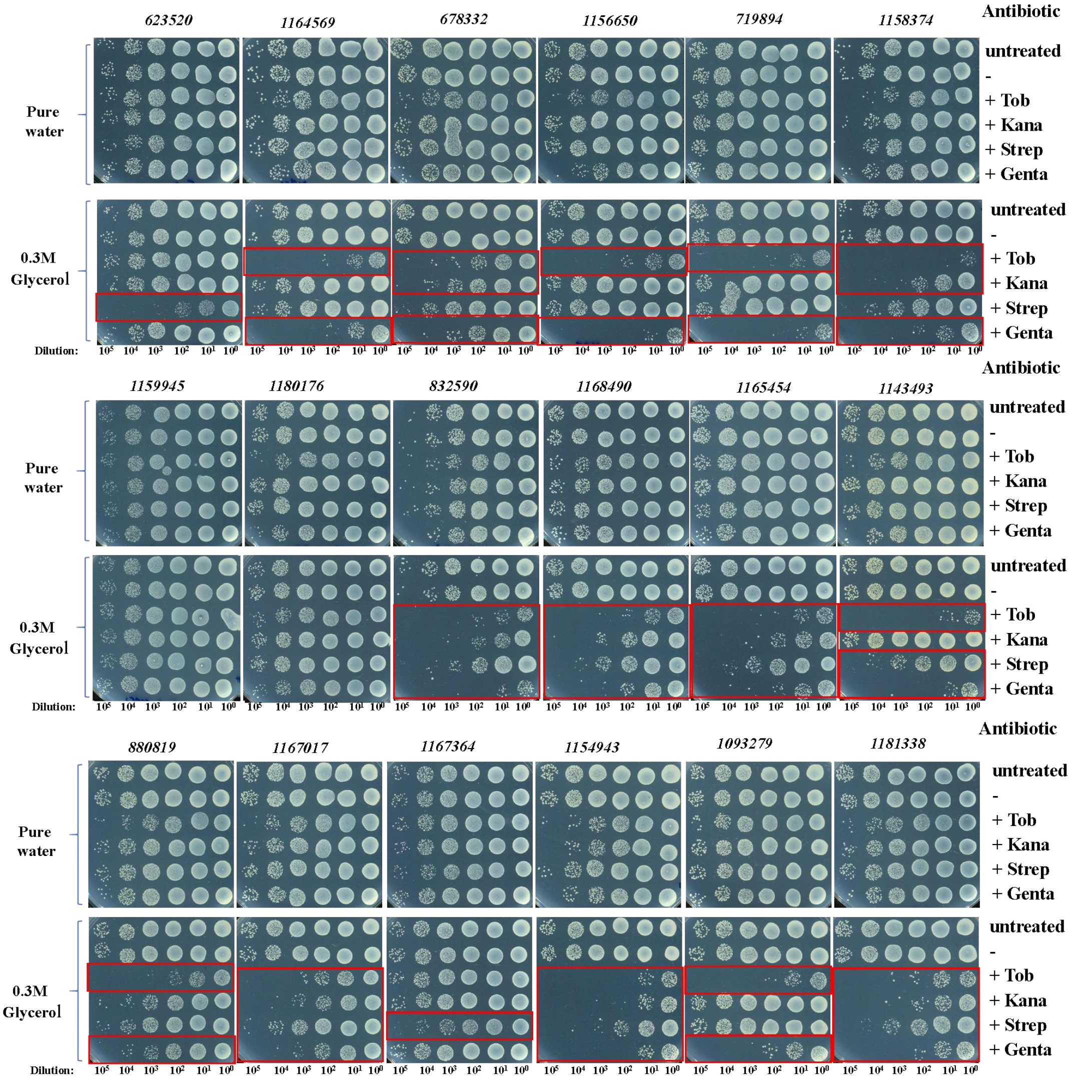

Figure S3 Glycerol potentiates aminoglycosides against clinical isolates of MRSA

Survival of 18 MRSA isolates (in stationary-phase) following a 3-min treatment with tobramycin, kanamycin, streptomycin or gentamicin plus 0.3 M glycerol

Figure S4

A

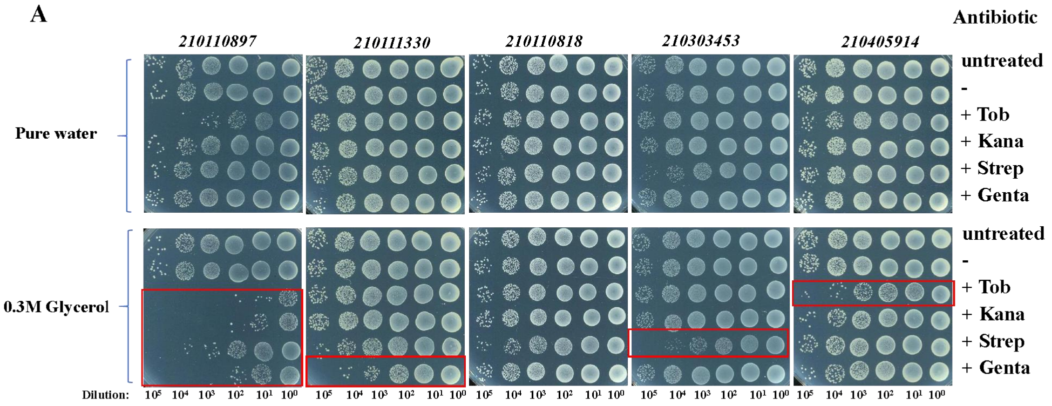

B

(μg/ml)	0	5	10	15	20	25	30	35	40	50	80	100
MRSA	0.585	0.518	0.451	0.338	0.454	0.329	0.295	0.339	0.36	0.025	0.006	0.005
MRSA	0.59	0.509	0.344	0.434	0.391	0.426	0.191	0.306	0.282	0.009	0.009	0.01
MRSA	0.712	0.571	0.396	0.478	0.382	0.485	0.613	0.342	0.417	0.071	0.016	0.009
Blank	0	0	0.001									
S. aureus	0.539	0.494	0.505	0.437	0.126	0.485	0.159	0.027	0.016	0.017	0.019	0.009
S. aureus	0.555	0.545	0.535	0.292	0.182	0.071	0.021	0.015	0.017	0.013	0.008	0.008
S. aureus	0.554	0.525	0.553	0.305	0.405	0.113	0.132	0.011	0.012	0.009	0.006	0.004

Figure S4 Glycerol potentiates aminoglycosides against clinical isolates of methicillin-sensitive *S. aureus*

(**A**) Survival of six methicillin-sensitive *S. aureus* isolates in stationary-phase following a 3-min treatment with tobramycin, kanamycin, streptomycin or gentamicin plus 0.3 M glycerol. (**B**) MIC assay for MRSA ATCC43300 and *S. aureus* ATCC29523 strains towards streptomycin.

Figure S5

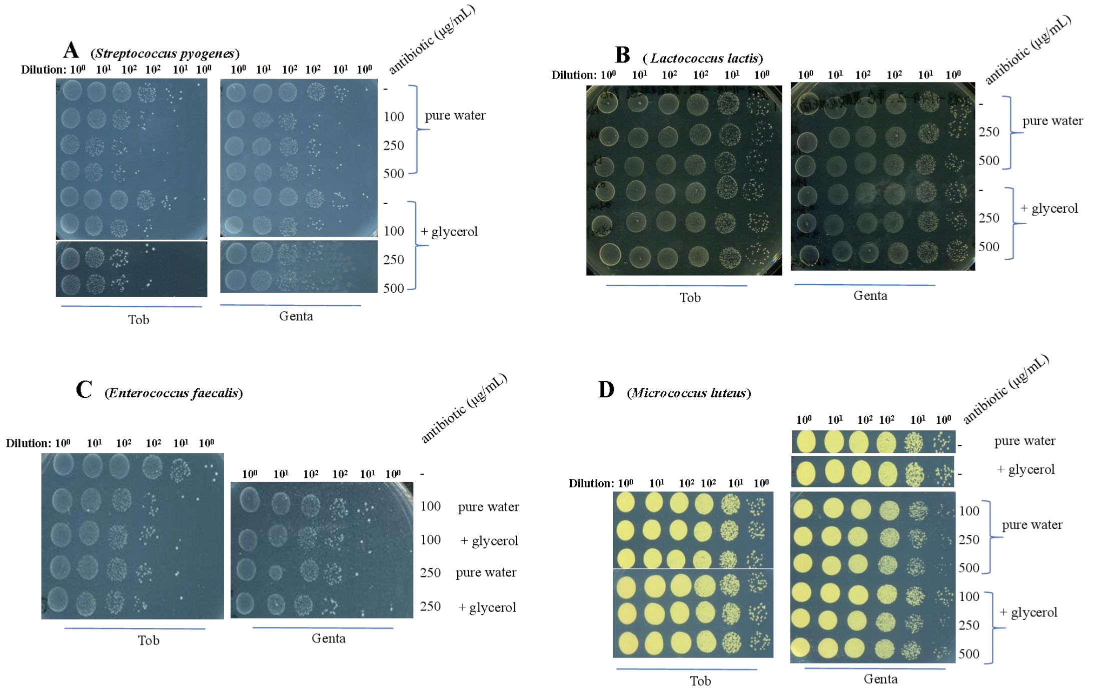

Figure S5 Glycerol has little potentiation effect on tobramycin and gentamicin against some pathogens

(**A, B, C, D**) Survival of indicated bacterial cells in stationary-phase following a 3-min treatment with tobramcyin and gentamicin at indicated concentrations as dissolved in pure water or 0.3 M glycerol solution. Panel A: *Streptococcus pyogenes*; panel B: *Lactococcus lactis*; panel C: *Enterococcus faecalis*; panel D: *Micrococcus luteus*.

Figure S6

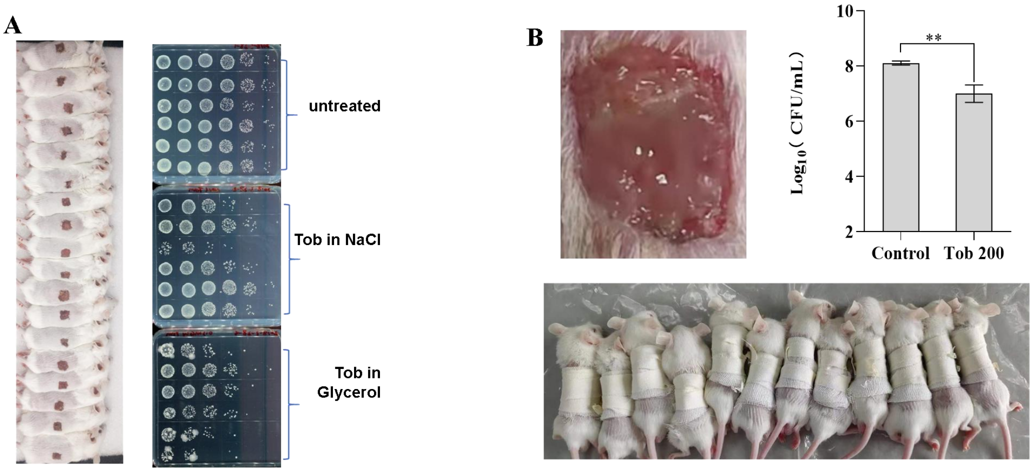

Figure S6 Glycerol potentiates tobramycin against *S. aureus* cells *in vivo*

(**A**) An acute skin wound model in mice. After bacterial seeding for 1 h and antibiotic treatment, mice were housed over-night (left part) and the whole muscle on the wound site was removed and homogenized, with the lysate being spot-plated on LB agar dishes for bacterial survival assay (right part). (**B**) *S. aureus* biofilm skin infection model in mice. Left: Imaging graphs of the *S. aureus* biofilm skin infection in mice. Lower: the skin wound was seeded with stationary-phase *S. aureus* cells (around 10^7 CFU), and then sealed with vaseline and wrapped with medical gauze. Right: survival of *S. aureus* biofilms after a 2-h treatment with 200 μg/m tobramycin.

Figure S7

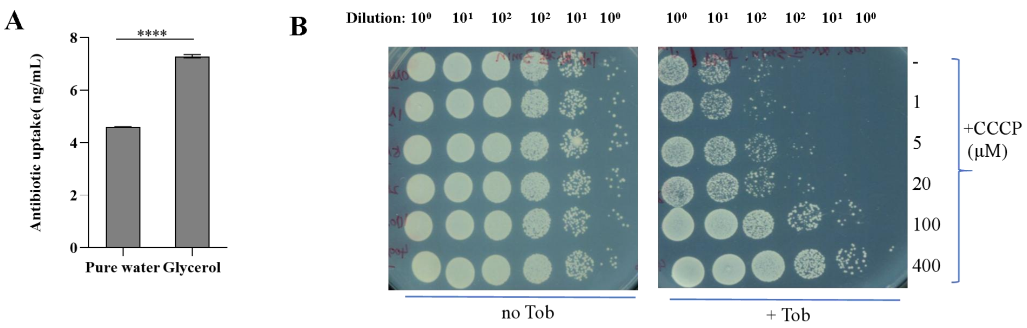

Figure S7 Glycerol enhances tobramcyin uptake in a PMF-dependent manner

(**A**) Quantified tobramycin uptake by *S. aureus* biofilms attached in a rubber catheter after a 15-min treatment with 100 μg/mL as dissolved in pure water or 0.3 M glycerol solution. (**B**) Survival of stationary-phase *S. aureus* cells following a 3-min treatment with 100 μg/mL tobramycin plus 0.3 M glycerol in the presence of increasing concentrations of CCCP.

Figure S8

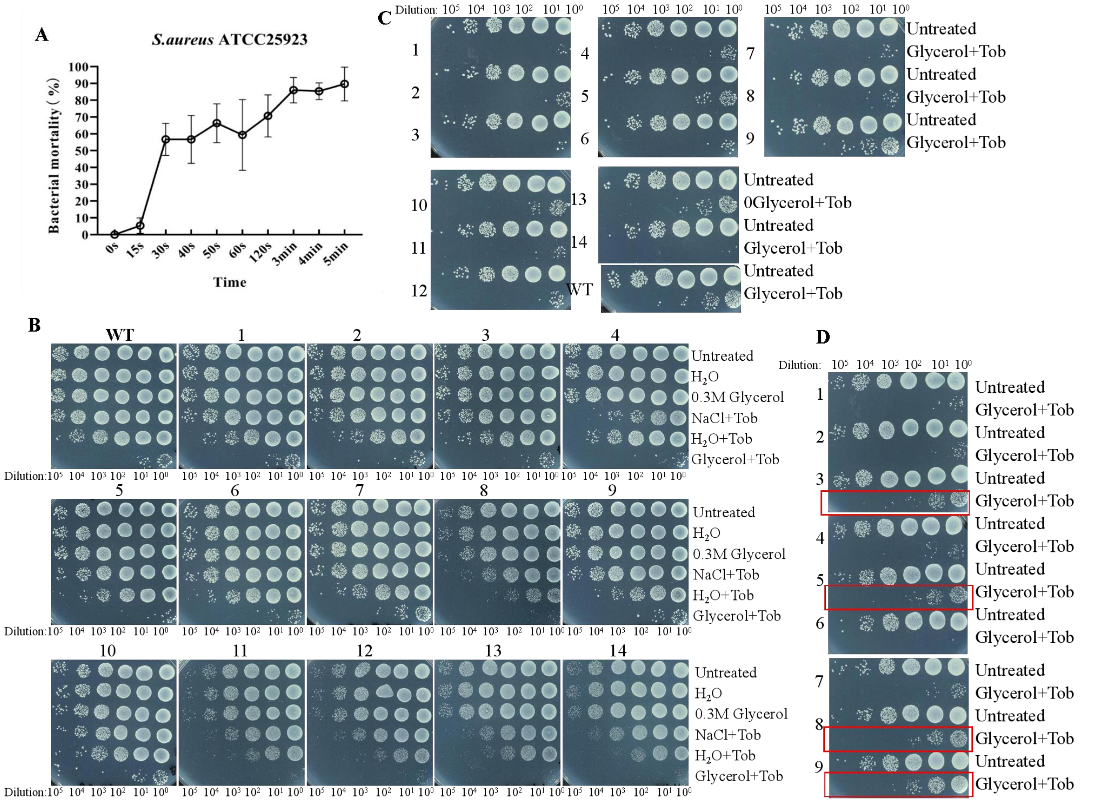

Figure S8 Isolation of *S. aureus* mutants tolerant to the combined treatment using ARTP-based library

(**A**) Time-dependent killing of *S. aureus* ATCC25923 cells following the treatment in the atmospheric and room temperature plasma (ARTP) instrument, with plasma radiation duration for mutagenesis being set as 3 min. (**B**) Survival of the fourteen *S. aureus* ARTP sub-libraries after one (panel B), two (panel C) and three (panel D) rounds of the combined treatment (50 μg/mL tobramycin plus 0.3 M glycerol) and cell culturing. Partial results for some sub-libraries after three rounds of treatment are shown in Fig. 5B.

Figure S9

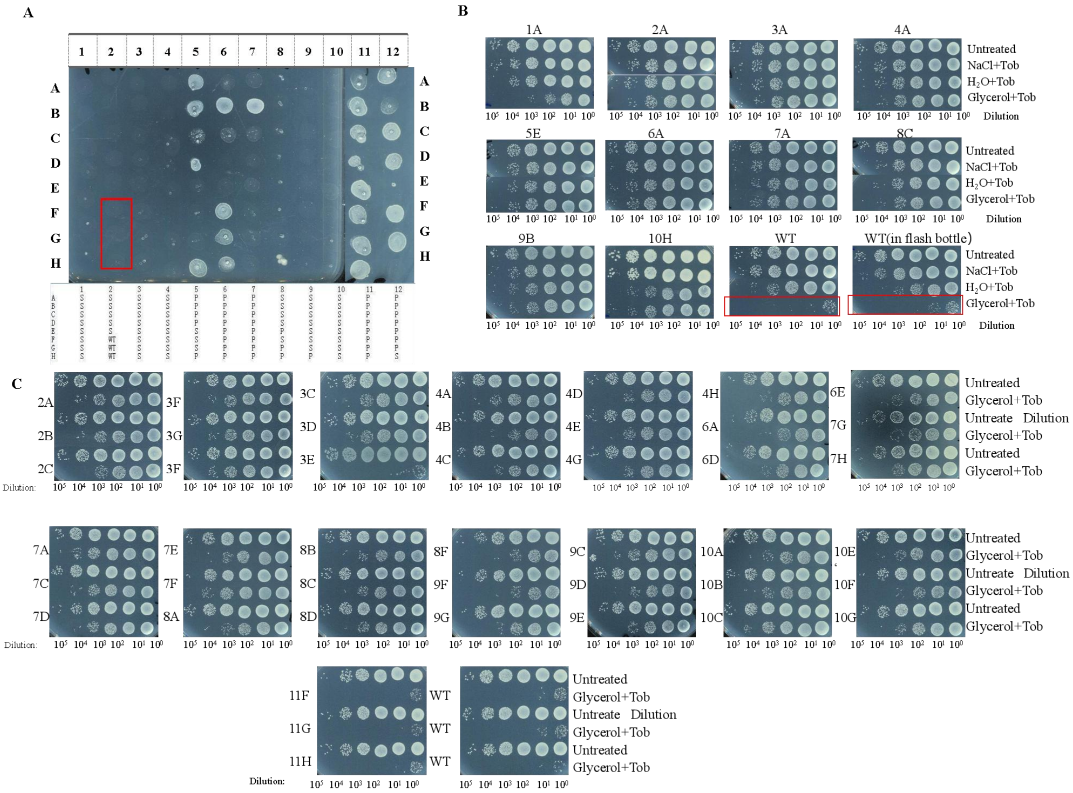

Figure S9 Validation of *S. aureus* tolerant clones

(A) Cell growth of *S. aureus* tolerant clones on the LB agar dish containing 2×MIC tobramycin (4 μg/mL). Red frame represents no growth of *S. aureus* wild type cells. (**B**) Survival of typical *S. aureus* tolerant clones as the first passage following a 3-min treatment with 50 μg/mL tobramycin as dissolved in pure water, 0.9% NaCl or 0.3 M glycerol solution. (**C**) Survival of typical *S. aureus* tolerant clones as the tenth passage following a 3-min treatment with 50 μg/mL tobramycin as dissolved in 0.3 M glycerol solution.

Figure S10

A

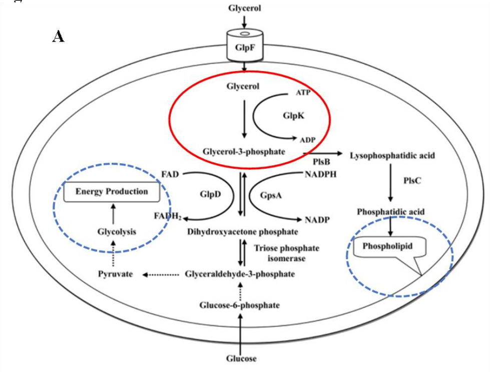

B

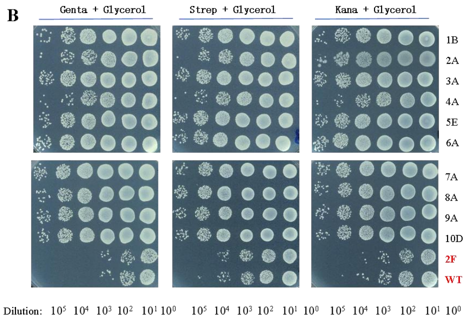

C

Name	Annotation	Impact	GeneName	Performance	Site
B1	frameshift_variant	HIGH	glpK	p. Ala439fs	1281887, C, CA
B9	missense_variant	MODERATE	glpK	p. Ser186Ile	1281131, G, T
C3	-	-	-	-	-
D3	stop_gained	HIGH	glpK	p. Gln234*	1281274, C, T
D6	stop_gained	HIGH	glpK	p. Trp198*	1281168, G, A
D9	missense_variant	MODERATE	glpK	p. Cys384Arg	1281724, T, C
E5	synonymous_variant	LOW	purH	p. Ala420Ala	1034458, T, C
	stop_gained	HIGH	glpK	p. Trp485*	1282029, G, A
E6	synonymous_variant	LOW	purH	p. Ala420Ala	1034458, T, C
	stop_gained	HIGH	glpK	p. Trp485*	1282029, G, A
F1	frameshift_variant	HIGH	glpK	p. Ala439fs	1281887, C, CA
F3	missense_variant	MODERATE	glpK	p. Gly243Asp	1281302, G, A
F9	missense_variant	MODERATE	glpK	p. Ser186Ile	1281131, G, T
	missense_variant	MODERATE	glpK	p. Cys384Arg	1281724, T, C
G1	frameshift_variant	HIGH	glpK	p. Ala439fs	1281887, C, CA
H7	stop_gained	HIGH	glpK	p. Trp463*	1281962, G, A
H9	missense_variant	MODERATE	glpK	p. Ser186Ile	1281131, G, T

D

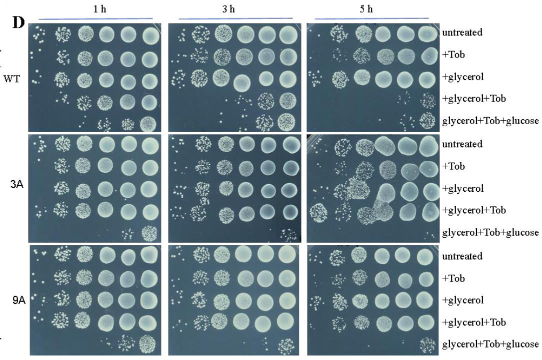

Figure S10 Characterization of *S. aureus* tolerant clones

(**A**) Glycerol-based energy and lipid metabolism pathways. Red circle represents GlpK-catalzyed conversion of glycerol to sn-glycerol 3-phosphate; blue dashed circle on the left represents energy metabolism as driven by glycerol; blue dashed circle on the right represents glycerol-derived phospholipids that eventually contributes to the biosynthesis of cell membranes. (**B**) Survival of typical *S. aureus* tolerant clones to the combined treatment with 50 μg/mL gentamicin, 100 μg/mL streptomycin or 100 μg/mL kanamycin plus 0.3 M glycerol. (**C**) Mutation sites in *S. aureus* tolerant clones. (**D**) Survival of ARTP-3A and ARTP-9A clones in stationary-phase following long-term treatment with 50 μg/mL tobramycin plus 0.3 M glycerol or glucose, which were directly added into the cell cultures.

Figure S11

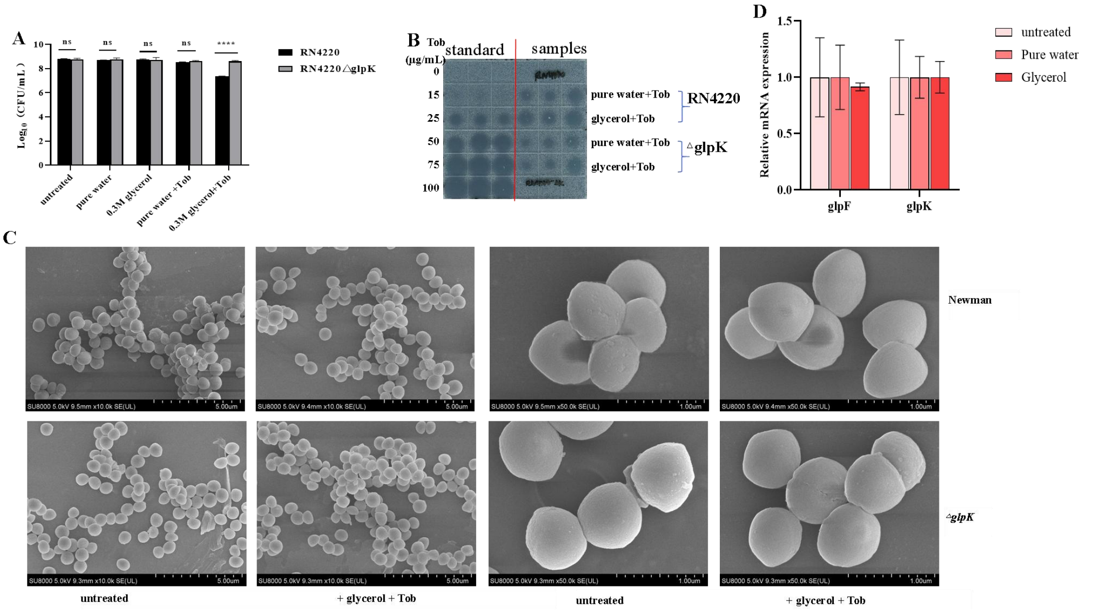

Figure S11 Effect of *glpK* on glycerol-induced tobramycin lethality

(**A**) Survival of *S. aureus* RN4220 wild type and *△glpK* mutant cells following the treatment with 50 μg/mL tobramycin as dissolved in pure water or 0.3 M glycerol solution. (**B**) Inhibition of *E. coli* cell growth on LB agar dishes by tobramycin extracted from stationary-phase *S. aureus* RN4220 wild type and △glpK mutant cells, which were pre-treated with 100 μg/mL tobramycin dissolved in pure water or 0.3 M glycerol. (**C**) Scanning electron microscopic graphs of *S. aureus* Newman wild type and *△glpK* mutant cells following the treatment with 50 μg/mL tobramycin plus 0.3 M glycerol. (**D**) Relative mRNA levels of *glpF* and *glpK* genes in *S. aureus* cells following 3-min treatment with 0.3 M glycerol or pure water, as revealed by RT-qPCR analysis.
